# Encoding Versus Linear Use of Patient Characteristics in Chest X-Ray Foundation Models on MIMIC-CXR

**DOI:** 10.3390/diagnostics16132030

**Published:** 2026-06-29

**Authors:** Yeonsu Kim, Yangwon Kim, Yoojin Nam, Namjoon Kim, Pa Hong

**Affiliations:** 1 Department of Radiology, Samsung Changwon Hospital, Sungkyunkwan University School of Medicine, Changwon 51353, Republic of Korea; yeonsu29.kim@samsung.com (Y.K.); yoojin8998.nam@samsung.com (Y.N.); 2 Department of Radiology and Research Institute of Radiology, Asan Medical Center, University of Ulsan College of Medicine, Seoul 05505, Republic of Korea; 3 Next Generation Semiconductor Convergence and Open Sharing System, Seoul National University, Room 609, Building 300, 1 Gwanak-ro, Gwanak-gu, Seoul 08826, Republic of Korea; knj01@snu.ac.kr; 4 Department of Digital Health, Samsung Advanced Institute for Health Sciences and Technology (SAIHST), Sungkyunkwan University, Seoul 06351, Republic of Korea

**Keywords:** chest X-ray, foundation model, attribute dependence, algorithmic fairness, odds ratio, residualization, shortcut learning, debiasing

## Abstract

**Background**: Chest X-ray (CXR) foundation models can predict patient demographic categories (sex, age, race) from images alone by linear probing, but whether encoded attributes drive finding prediction has not been tested at scale. **Methods**: On MIMIC-CXR (230,697 images, 60,518 patients), we measured attribute dependence (AUROC drop after residualizing an attribute from a frozen embedding) across 24 patient attributes (four demographics and 20 ICD-coded comorbidities), 10 thoracic findings, and 6 overlap-free foundation models (n=1440 triplets), with 3 additional CXR-pretrained models (RAD-DINO, CheXzero, CheSS) for encoding and fairness analyses. Dependence was regressed on attribute-finding odds ratios (ORs), encoding strength, and model-level factors. **Results**: Encoding and dependence dissociated. Sex (AUROC 0.942) contributed <0.001; race (0.83) contributed 0.0015 (rank 14/24); heart failure (0.774) showed the largest dependence (0.018). |log(OR)| explained 50.6% of dependence variance (β=0.029, $p<10^{-15}$); model factors added no detectable contribution ($\Delta R^{2}=0.000$, n=6). Residualizing the top three high-OR attributes reduced AUROC by 0.026 without narrowing sex or age subgroup gaps (minimum detectable effect size (MDES) = 0.0019). Across 9 models, four-category race subgroup gaps (mean 0.069) were 30–75× larger than race residualization drops (mean 0.0015); CheXzero showed the same decoupling. **Conclusions**: Encoding, residualization-sensitive dependence, and subgroup bias are three separable quantities on the same model. Pre-deployment audits on inpatient-skewed cohorts can prioritize attributes by local OR; jointly residualizing race and its cardiac correlates does not narrow the race subgroup gap, which instead tracks group-wise finding base rates. Cross-institutional transfer remains open: no public CXR cohort currently links comorbidity electronic health records for external validation of the OR-dependence relationship.

## 1. Introduction

Foundation models trained on chest X-ray (CXR) images can predict patient demographic categories such as sex, age, and race from imaging features alone [1,2]; here and throughout, this refers to classifying demographic categories from frozen embeddings (linear probing), not to reconstructing patient-identifying information. This finding has anchored a research agenda in medical-AI fairness around an implicit three-step pipeline: a foundation model encodes protected attributes from the image, the encoded attributes bias finding prediction, and the biased prediction produces subgroup performance gaps. Debiasing methods intervene at the first step by stripping demographic information from learned representations [3]; shortcut-learning studies reinforce the pipeline by showing that models can exploit spurious imaging features unrelated to the disease label [4,5].

The transition from the first to the second step has received less direct evidence. Glocker et al. [2] measured how strongly several attributes were encoded but did not test whether the encoded signal drove finding prediction. Brown et al. [6] proposed a framework for detecting shortcut learning but did not compare the magnitude of shortcuts across multiple attributes. Subgroup audits to date [7,8,9,10] report performance gaps across models without separating two candidate drivers: attribute-finding clinical associations versus model-level factors such as architecture, learning regime, or pretraining domain. Recent work also documents that debiasing interventions that close subgroup gaps in one cohort do not always transfer to another [11], motivating audits that explicitly account for cohort-level attribute-finding structure.

We address this question within a single cohort by separating three quantities on the same frozen embedding: encoding (can an attribute be decoded from the embedding?), residualization-sensitive dependence (does finding AUROC drop when the attribute signal is linearly removed?), and subgroup bias (does the resulting predictor have unequal per-group AUROC?). Attribute dependence is measured as the AUROC drop after residualizing a given attribute from the embedding: a positive value indicates the prediction was statistically dependent on that attribute under linear residualization, and a value near zero indicates the attribute is detectable but that removing its linear signal does not change the prediction. We cover 24 patient attributes (four demographics plus 20 ICD-coded comorbidities), 10 thoracic findings, and nine foundation models that span three training paradigms (supervised, self-supervised, vision-language), three architecture families (CNN, vision transformer, ConvNeXt), and three pretraining domains (natural images, biomedical images, CXR). Six overlap-free models anchor all residualization analyses (ResNet50-ImageNet, DINOv2-base, BiomedCLIP, XRV-DenseNet-nih, CLIP-ViT-B16, and ConvNeXtV2-Base), and three CXR-specialized models with pretraining overlap (RAD-DINO, CheXzero, and CheSS) are included for encoding and fairness analyses. One of these, CheXzero (the driving example in Yang et al. [10]), is carried through all three quantities as a descriptive within-cohort check; because it shares the MIMIC cohort, this is not an independent replication.

Against dependence we compute attribute-finding odds ratios (ORs) from electronic health record (EHR)-linked comorbidity labels and ask which signal best tracks dependence, and whether residualizing the highest-OR attributes narrows subgroup fairness gaps. The three quantities dissociate: encoding strength is a poor predictor of residualization-sensitive dependence; attribute-finding clinical associations track dependence closely, with no detectable contribution from model-level properties in our sample; and per-model race dependence does not covary with per-model race subgroup AUROC gap (DINOv2-base, tied with CLIP-ViT-B16 for the highest race dependence at 0.0018, has the smallest race gap at 0.056, while XRV-DenseNet-nih, with the lowest race dependence at 0.0011, has one of the largest gaps at 0.083). These observations motivate a shift in how pre-deployment fairness audits prioritize attributes, which we develop in Section 4.1.

## 2. Materials and Methods

### 2.1. Data

This was a multi-model retrospective observational study. MIMIC-CXR v2.0 [12,13] provided 230,697 frontal chest radiographs from 60,518 patients at Beth Israel Deaconess Medical Center (2011–2016), a cohort skewed toward intensive-care and emergency admissions (60.5% portable anteroposterior). Cohort demographics and comorbidity prevalence are summarized in Table 1. Mean age was 56.9±19.5 years, 52.2% female; body mass index (BMI) was available for 70.4% of patients (78.7% of images, reflecting that patients with recorded BMI tended to have more imaging studies). Finding labels came from the CheXpert labeler [14] on free-text reports under the U-Ignore policy (uncertain and unmentioned labels masked, not treated as negative), restricting analysis to definitive positive/negative readings. We excluded two findings with extreme class imbalance (Fracture, Lung Lesion) and two meta-labels (No Finding, Pleural Other), leaving 10 thoracic findings: Atelectasis, Cardiomegaly, Consolidation, Edema, Enlarged Cardiomediastinum, Lung Opacity, Pleural Effusion, Pneumonia, Pneumothorax, and Support Devices. Support Devices is the CheXpert labeler’s marker for the presence of tubes or lines (not a pathology); we retained it at the labeler’s native label granularity to keep our analysis comparable to prior CheXpert-based audits [7,14]. Per-finding valid-label counts ranged from 12,448 to 81,015 images.

Patient attributes (24) combined four demographics (age, biological sex, BMI, race) with 20 International Classification of Diseases (ICD)-code comorbidities drawn from MIMIC-IV [15,16]: heart failure, atrial fibrillation, coronary artery disease, chronic kidney disease, diabetes, chronic obstructive pulmonary disease (COPD), obesity, hypertension, hyperlipidemia, anemia, and ten others (Table S1). Race was obtained from the MIMIC-IV admissions.race field (per-patient majority non-Unknown category) and collapsed to four groups (White, Black, Asian, Other). For residualization and OR calculation we used a binary Black-vs-White contrast (Asian and Other excluded for that specific attribute, leaving 182,084 images from 40,816 patients; 19.6% Black), which matches the primary metric used in prior CXR race-encoding work [1]; all four race groups were retained for the subgroup fairness analysis in Section 3.4. Each MIMIC-CXR imaging study was linked to MIMIC-IV at the patient level through subject_id; ICD codes were aggregated across all of a patient’s MIMIC-IV admissions and a comorbidity was coded as present if the corresponding ICD appeared on any admission (patient-level ever-coded definition). Because MIMIC-IV assigns diagnosis codes at discharge rather than at the time of imaging, some codes reflect conditions confirmed after the index CXR; we treat this as a measurement-timing caveat in Section 4.2 and report the patient-level OR in the same direction as the image-level analysis as a sensitivity check (Table S7). Missingness for ICD-coded comorbidities was zero by design (absence of code equals absence of diagnosis under this definition). We split at the patient level with sex-stratified 70/15/15 ratios (random patient allocation, not time-ordered, following convention in prior CXR fairness audits [7,9]) to prevent leakage.

### 2.2. Foundation Models

We selected six models spanning a 3×3 (training paradigm × pretraining domain) taxonomy, with no overlap between pretraining and evaluation data (Table 2). The clean-model panel fills five of the nine cells: supervised–natural (ResNet50-ImageNet), self-supervised–natural (DINOv2-base and ConvNeXtV2-Base; two models sampled to strengthen this cell’s power for the model-level contribution test in Section 3.3), vision-language–natural (CLIP-ViT-B16), vision-language–biomedical (BiomedCLIP), and supervised–CXR (XRV-DenseNet-nih, trained on NIH ChestX-ray14, which has no patient overlap with MIMIC-CXR). Three additional CXR-specialized models—RAD-DINO (self-supervised–CXR), CheSS (supervised–CXR with MIMIC-ancestral exposure), and CheXzero (vision-language–CXR)—were pretrained on datasets that overlap with MIMIC-CXR and are therefore reserved for encoding and fairness analyses only (Section 2.2 paragraph below on overlap models). MedCLIP and PubMedCLIP were excluded because their pretraining corpora include MIMIC-CXR reports. ViT-L-scale variants were not included due to compute constraints; the six clean models span a 20-fold effective-rank range (5.6 for XRV to 109.4 for ResNet50-ImageNet, Table 2) and cover three training paradigms, providing the variance needed for the model-level contribution test.

Three CXR-specialized models with pretraining–evaluation overlap (RAD-DINO [23], CheXzero [24], CheSS [25]) were excluded from the primary OR→dependence residualization analyses; they enter the encoding and fairness analyses, with CheXzero additionally carried through per-attribute race dependence as a descriptive within-cohort check (Table S13). Images were resized to each model’s native resolution, normalized with model-specific statistics, and passed through frozen networks. We took the final-layer embedding (global average pooling for CNNs, CLS token for ViTs) and standardized to zero mean and unit variance using training-set statistics.

### 2.3. Residualization, Dependence, and Odds Ratios

**Attribute dependence.** For each (attribute, model, finding) triplet we (i) fit an L2-regularized (ridge, α=1) linear regression of the standardized training embeddings *X* on the attribute *a*; (ii) subtracted the fitted values to form residualized embeddings $\tilde{X}=X-\hat{X}\left(a\right)$, removing the component of the embedding linearly predictable from the attribute; (iii) retrained an L2-regularized logistic regression classifier on $\tilde{X}$, holding the regularization constant fixed across original and residualized runs; (iv) computed attribute dependence as $\mathrm{AUROC}\left(X\right)-\mathrm{AUROC}\left(\tilde{X}\right)$. Dependence is an *associational* quantity—the change in the linear probe’s predictive performance when the attribute’s linear signal is removed—not a causal-mediation estimate: a positive value means the probe’s predictions are statistically dependent on the (linearly encoded) attribute, and does not establish that the model causally relies on the attribute as such rather than on correlated imaging features. A value near zero means the attribute is detectable in the embedding but its linearly-predictable component does not contribute to the prediction (an encoding–linear-use dissociation). Because ridge residualization removes only the (regularized) linear component of *a*, dependence quantifies *linear* use specifically; nonlinearly-encoded attribute information may survive, which we probe directly in Section 2.4 and discuss in Section 4.3.

Attribute-level dependence is the mean across model × finding cells with bootstrap 95% confidence interval (CI) from 10,000 resamples. We use a linear residualization throughout, in keeping with standard probing methodology in the analysis of neural representations [26].

**Figure 1:**
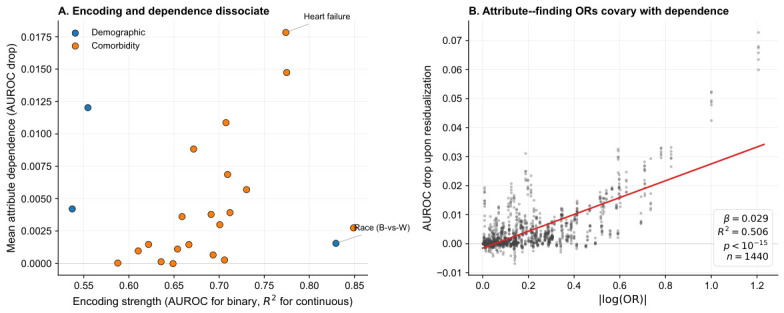
**Encoding strength and attribute dependence dissociate; attribute-finding odds ratios covary with dependence.** (**A**) Encoding strength (*x*) versus mean attribute dependence (*y*) for 24 patient attributes (one point per attribute); demographics in blue, comorbidities in orange. Sex is encoded at AUROC 0.942 yet contributes essentially zero, while heart failure (encoding 0.774) shows the largest dependence. (**B**) |log(OR)| versus AUROC drop across all 1440 model-attribute-finding observations (one grey point per observation) (β=0.029, $R^{2}=0.506$, $p<10^{-15}$); the six clean models overlap on a shared regression line. Methodological detail (binarization, bootstrap) in Section 2; full per-model decomposition in Table S1.

**Encoding strength.** L2-regularized linear probes on frozen embeddings reported AUROC for 22 binary attributes (20 comorbidities plus sex and Black-vs-White race) and ridge $R^{2}$ for 2 continuous attributes (age, BMI). For the scale-unified encoding–dependence scatter in Figure 1A, continuous attributes were median-binarized and re-probed as binary for AUROC; native $R^{2}$ values appear in Table S1.

**Odds ratios.** For each attribute-finding pair we computed the image-level odds ratio from a 2 × 2 contingency table on the MIMIC-CXR training set, applying a 0.5 continuity correction to all four cells (uniformly, not only when a cell was zero) for numerical stability across the 24 × 10 OR matrix. Continuous attributes were binarized at the median. We chose the image-level unit as the primary measure because the dependent quantity in our regression—the AUROC drop—is itself estimated per image on the same test set; computing the OR on the same unit avoids a unit mismatch between predictor and outcome and keeps both on the population the classifier actually sees. Because the image level over-weights frequently-imaged (and typically sicker) patients, we verify the choice does not drive the result with two sensitivity analyses that preserve the attribute ranking: patient-level odds ratios (one random image per subject, n=60,518; Spearman ρ=0.76 with image-level; Table S7) and inverse-probability-weighted odds ratios (w=1/images_per_patient; ρ=0.963; Table S9). The full 24 × 10 OR matrix appears in Table S4.

### 2.4. Statistical Analysis

The primary test was a nested OLS regression on 1440 observations (24 attributes × 10 findings × 6 clean models). Predictors of attribute dependence were |log(OR)|, encoding strength, log(effectiverank), model identity, and their interactions. Because the same odds ratio applies to all six models, observations are not independent, so we corroborated the OLS estimate with three corrections: attribute-clustered (24) and finding-clustered (10) robust standard errors, and a mixed-effects model with random finding intercepts. A collapsed cell-level regression (n=240) gave independent confirmation.

For testing encoding as a covariate (specification M1a), we restricted to the 22 binary attributes (n=1320), so that encoding was uniformly an AUROC.

Circularity between OR and dependence being measured on the same cohort was addressed by leave-one-finding-out (LOFO) and leave-one-attribute-out (LOAO) cross-validation of the OR → dependence regression, with $R_{\mathrm{OOS}}^{2}$ on held-out units. To probe nonlinearity we ran two checks (Table S18). First, the dependence *ranking* is robust to nonlinear residualization: for the two continuous attributes (the only non-degenerate case—for binary attributes the MLP map reduces to the linear group-mean map), a 2-layer MLP residualization left the 24-attribute ranking unchanged (Spearman ρ=1.00). Second, the dependence measure targets the *linear* probe used throughout: ridge residualization removes the attribute’s linearly-predictable component from the embedding, so the AUROC drop captures the linear finding classifier’s reliance on the attribute’s linear signal—the frozen-embedding, linear-probe regime that the encoding and linear-debiasing literature targets. The removal is, however, explicitly *linear*: an MLP still decodes sex and race from the residualized embedding (AUROC 0.97 and 0.91), so distributed nonlinear demographic structure is not erased. We therefore report dependence as a measure of *linear* use (Section 4.3). Third, to test the hierarchy directly under nonlinear removal, we erased five representative attributes (heart failure, atrial fibrillation, age, race, and sex) by iterative nullspace projection in a random-Fourier-feature kernel space (nonlinear in the embedding) with a matched random-direction control (Table S19): the attribute ranking was identical to the linear ordering (Spearman ρ=1.0; heart failure and age highest, sex and race the two lowest, ∼5-fold lower than heart failure), so the hierarchy is *not* a linear-residualization artifact, while demographics retained a small non-zero nonlinear residual consistent with the linear-use scope. XRV-DenseNet-nih’s MLP residualization diverged at its very low effective rank (5.6; MLP $R^{2}=-154.6$) and was excluded from the MLP comparison only.

Cross-model consistency was assessed by pairwise Spearman rank correlations of attribute-level dependence across all 15 model pairs.

The fairness test jointly residualized the top three high-OR attributes (heart failure, atrial fibrillation, age) and measured subgroup AUROC gaps (by sex, and by age tertiles of <50, 50–70, and ≥70 years) across all 10 findings for the 6 clean models, yielding n=120 model × finding × dimension cells. Three findings with >90% prevalence (Atelectasis, Lung Opacity, Support Devices) have less stable subgroup AUROCs and are retained in the main analysis; per-model gaps for these findings are reported in Table S3 alongside the other 7 findings. The effective-rank-versus-gap association analysis expanded to all 9 models (adding RAD-DINO, CheXzero, and CheSS), because fairness concerns post-deployment behavior regardless of pretraining source. MLP classifiers used three random seeds, and permutation tests of significance were corrected by Benjamini-Hochberg false discovery rate (FDR).

We frame a single primary hypothesis (the OR → dependence regression on the full 1440-triplet panel); per-triplet multiple-testing correction is not applicable because individual triplets are not tested as separate hypotheses. Residual diagnostics indicated a heavy-tailed, heteroscedastic distribution (skewness 1.68; Breusch-Pagan $p<10^{-15}$), so we do not use parametric OLS standard errors for inference. Reported significance rests on attribute- and finding-clustered robust SEs, percentile bootstrap CIs (2000 replicates), and a Huber M-estimator sensitivity check (Table S11); all three agreed with the OLS point estimate. Multicollinearity among comorbidities was modest (all variance inflation factors (VIFs) <2; maximum pairwise |r|=0.461). Full regression specifications, bootstrap schemes, effective-rank definition, and MLP architecture appear in Supplementary Methods.

### 2.5. Ethics and Reproducibility

Samsung Changwon Hospital IRB approved exempt status (protocol code SCMC 2026-03-007, approved 23 March 2026; Declaration of Helsinki). All datasets are publicly available under credentialed data use agreements; no patient re-identification was attempted.

Python 3.11, PyTorch 2.1, scikit-learn 1.4, statsmodels 0.14; NVIDIA RTX 5090 GPU; seeds fixed (42 for splits, 0/1/2 for MLP ensembles). Analysis code and trained probe weights will be released on a public GitHub repository upon acceptance.

Generative artificial intelligence tools were used only for language editing and formatting suggestions during manuscript preparation; all scientific content, analyses, and interpretations are the authors’ own.

## 3. Results

### 3.1. Encoding and Dependence Dissociate

Figure 1A plots encoding strength against attribute dependence across the 24 patient attributes; per-finding baseline AUROCs are reported in Table S8. The two quantities dissociated. Sex was the most strongly encoded attribute (AUROC 0.942) but contributed essentially zero to finding prediction (mean drop <0.001, 95% CI [−0.000, 0.001]). Black-vs-White race was encoded at AUROC 0.71–0.90 across the six models (mean 0.83) yet contributed only 0.0015 on average, reproducing the encoded-but-weakly-dependent pattern at a second socially protected attribute. At the opposite pole, heart failure was encoded less well (0.774) yet showed the largest dependence (0.018, 95% CI [0.013, 0.023]). The top five attributes by dependence were all clinical comorbidities or age (heart failure, atrial fibrillation, age, acute kidney injury, anemia; Table 3); the bottom of the ranking was populated by attributes the embeddings detected but on which predictions did not depend under linear residualization (sex, asthma, depression, smoking history; all |drop|<0.001).

Encoding and dependence were only weakly rank-correlated at the attribute level. Spearman ρ was below 0.3 and non-significant across the full 24 attributes (mixing AUROC and $R^{2}$), and near 0.5 when restricted to the 22 binary attributes with a unified AUROC metric—statistically significant, but a far smaller effect than the OR-dependence relationship quantified below.

### 3.2. Attribute–Finding Odds Ratios Covary with Dependence

Figure 1B plots |log(OR)| against AUROC drop for all 1440 observations (24 attributes × 10 findings × 6 models). A single regression line explained 50.6% of dependence variance (β=0.029, $p<10^{-15}$, n=1440). Adding finding intercepts raised $R^{2}$ to 61.5%, and adding attribute intercepts raised it further to 77.7% (Table 4). On the binary-attribute subset (n=1320), encoding strength added a small but significant increment beyond OR (specification M1a: $\Delta R^{2}=0.006$, p<0.001), two orders of magnitude smaller than the OR main effect.

The OR effect survived all three non-independence corrections: attribute-clustered robust SE ($p=8.5\times 10^{-7}$), finding-clustered ($p=4.9\times 10^{-3}$), and mixed effects with random finding intercepts ($p<10^{-15}$). Collapsing to the attribute-finding cell level (n=240) yielded β and $R^{2}$ essentially unchanged. Because the image-level unit over-weights frequently imaged (often sicker) patients, we ran two patient-weighting sensitivity analyses: inverse-probability-weighted OR (w=1/images_per_patient) preserved the attribute ranking (ρ=0.963 with the unweighted ranking) and produced a cell-level $R^{2}=0.675$ (vs. 0.734 unweighted), and patient-level OR (one random image per subject) showed the same effect direction (Tables S7 and S9); the OR-dependence relationship is therefore not an artifact of repeated imaging.

The two cross-validation tests separated direction from magnitude. Within-finding Spearman ρ between |log(OR)| and dependence was positive in 9 of 10 findings (Table S2, Figure S2), with Atelectasis as the sole exception (ρ=−0.238, attributable to its extreme class imbalance at 0.97 positive prevalence). Leave-one-attribute-out cross-validation on held-out attributes yielded $R_{\mathrm{OOS}}^{2}=0.459$. Leave-one-finding-out cross-validation, which tests whether the slope of the regression generalizes to a new finding, gave positive $R_{\mathrm{OOS}}^{2}$ for 6 of 10 findings (best: Edema 0.844, Pneumonia 0.713) and was worse than a constant-mean predictor for the other four. The direction of the OR-dependence relationship generalized; the absolute slope did not.

### 3.3. No Detectable Model-Level Contribution Within the Tested Model Set

**Figure 2:**
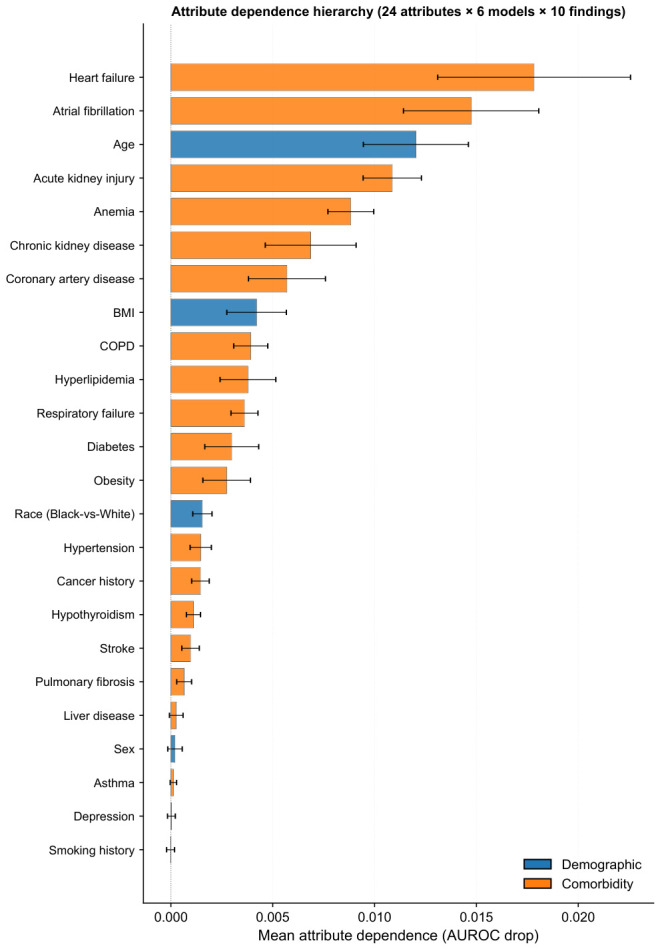
**Attribute dependence hierarchy across 24 patient attributes.** Waterfall chart of mean AUROC drop upon residualization (mean across 6 foundation models × 10 thoracic findings); error bars show bootstrap 95% CIs from 10,000 resamples. Demographic attributes (blue) and comorbidities (orange). Top-ranked attributes are clinical comorbidities and age; sex clusters near zero despite its AUROC 0.942 encoding. Full per-model decomposition in Table S1.

All 15 pairwise Spearman rank correlations of attribute-level dependence across the six clean models exceeded 0.96 (range 0.969–0.995; mean 0.981; Table S10). The top five attributes were identical across CNN, ViT, and ConvNeXt architectures, across supervised, self-supervised, and vision-language objectives, and across a 20-fold range in effective rank (5.6 for XRV-DenseNet-nih to 109.4 for ResNet50-ImageNet). The full ranking appears in Figure 2. Per-model OR → dependence slopes were tightly clustered (range 0.029–0.033; Figure S1), with within-model Spearman correlations between |log(OR)| and dependence of ≥0.525 for every model ($p<10^{-17}$). The within-model correlation (0.525) and between-model rank correlation (0.96) measure distinct quantities: the former asks whether higher-OR attributes have higher dependence within a single model, while the latter asks whether the attribute ranking itself transfers across models. Both held, consistent with an attribute hierarchy that is preserved across models and tracks OR within each.

The nested regression returned the same answer by null-hypothesis test. Adding log(erank) (p=0.665), model identity (p=0.907), or their interactions with OR (p=0.803 and 0.905) did not improve fit beyond OR and finding intercepts (all $\Delta R^{2}=0.000$, n=6). Post-hoc power was limited at six models: model-level predictors contribute df = 1 (continuous log(erank)) to df = 5 (model identity) over an effective n=6, so the minimum detectable $\Delta R^{2}$ under Cohen’s convention for a small effect [27] is approximately 0.02 at α=0.05 and 80% power; smaller model-level contributions cannot be ruled out by this test. The two-model filling of the self-supervised–natural cell (DINOv2-base, ConvNeXtV2-Base) slightly improves within-cell variance estimation for that cell without changing the n=6 model-identity df. Extending the regression to a nine-model panel by adding the three overlap models (RAD-DINO, CheXzero, and CheSS; n=2160 observations on the 24-attribute panel) gave qualitatively identical conclusions: log(effective rank) added no detectable variance beyond OR and finding intercepts ($\Delta R^{2}=0.000$), model identity contributed $\Delta R^{2}\approx 0.002$, and the strongest interaction (OR × model identity) contributed $\Delta R^{2}\approx 0.007$ (Table S12).

### 3.4. Residualizing High-OR Attributes Does Not Reduce Sex or Age Subgroup Gaps

**Figure 3:**
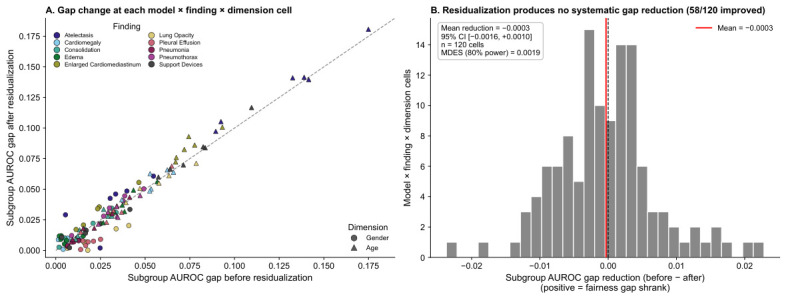
**Residualizing the top-OR attributes reduces AUROC without reducing subgroup gaps.** Subgroup AUROC gap reduction (before − after, positive = gap shrank) across 6 models × 10 findings × 2 subgroup dimensions (sex, age tertiles) for the 120-cell test. Mean reduction −0.0003 (95% CI [−0.0016, 0.0010]); 58/120 (48.3%) cells improved; MDES at 80% power =0.0019. Individual findings move in both directions (Pleural Effusion 0.018 → 0.006; Atelectasis 0.031 → 0.038). In panel (**A**), the grey dashed line is the identity (y=x); in panel (**B**), the black dashed line marks zero reduction and the red line marks the mean reduction (−0.0003).

Joint residualization of the top three high-OR attributes (heart failure, atrial fibrillation, age) across six models and 10 findings reduced overall AUROC by 0.026 but produced no systematic change in sex or age subgroup gaps. The mean gap reduction was −0.0003 (95% CI [−0.0016, 0.0010]) across 120 model × finding × dimension cells, with 58/120 (48.3%) cells showing any improvement. Individual findings moved in both directions (Figure 3): the Pleural Effusion gap contracted from 0.018 to 0.006, while the Atelectasis gap widened from 0.031 to 0.038. The 95% CI half-width corresponds to a minimum detectable effect size (MDES) of 0.0019 at 80% power (α=0.05, two-sided), ruling out systematic gap reductions larger than approximately 0.002 (smaller effects remain plausible, and the MDES would be slightly larger under cell clustering).

Extending the analysis to all nine models (adding the overlap models RAD-DINO, CheXzero, and CheSS) gave the same picture. Model-level representational geometry (log effective rank) did not covary with gap magnitude (sex: ρ=0.143, p=0.760; age: ρ=−0.214, p=0.645). A nominal sex-gap-versus-erank association appeared when we controlled for finding (p=0.044 uncorrected, higher erank → larger gap, opposite to prior speculation) but would not survive FDR correction across the multiple sub-tests summarized in Table S3, and we treat it as descriptive pending replication at larger model counts. Because AUROC alone does not fully characterize fairness, we additionally report per-subgroup sensitivity, specificity, false-positive and false-negative rates, and calibration error at the Youden operating point, with bootstrap 95% CIs for every subgroup gap (Table S21). The operating-point gaps are larger than the AUROC gaps (e.g., age and race specificity/FPR gaps of 0.14–0.17 versus AUROC gaps of 0.05–0.07), and all AUROC-gap CIs exclude zero (e.g., race 0.069, 95% CI [0.027, 0.183]); the subgroup gaps are thus robustly present across metrics, which is distinct from—and does not contradict—the finding that demographic residualization does not detectably *reduce* them.

### 3.5. Demographic Output Gaps and Demographic Dependence Are Decoupled

The four-category race dimension (White, Black, Asian, Other; ≥30 images per group required) revealed a dissociation in the opposite direction from the sex and age result above. Mean four-category race subgroup AUROC gap across 90 model–finding cells was 0.069 (range 0.056 for DINOv2-base to 0.085 for ResNet50-ImageNet)—approximately twice the mean sex-and-age subgroup gap (0.036 across 120 sex- and age-stratified cells, Section 3.4)—while race residualization-sensitive dependence on the same models was 30–75× smaller (mean 0.0015; Table S13). CheXzero (the driving example in the recent vision-language foundation-model demographic-bias evaluation of Yang et al. [10]) showed the same pattern on our cohort: race dependence 0.0013 alongside a race subgroup gap of 0.059 (maximum 0.122 for Atelectasis). Because CheXzero shares the MIMIC pretraining data, this is a descriptive within-cohort check rather than an independent replication.

Residualization-sensitive demographic dependence and demographic-stratified output disparity are therefore empirically decoupled on the same model and cohort: a model can produce demographically uneven output without being describable as “using” demographics as a linear predictor in the sense captured by our residualization. The MDES bound of the fairness test (0.0019; Section 3.4) implies we can rule out demographic-residualization-driven reductions of the 0.069 race subgroup gap larger than ∼3% (∼0.002 absolute AUROC); smaller reductions cannot be excluded.

Race was scoped out of the top-three high-OR residualization set in Section 3.4 because its OR was dominated by clinical comorbidities. We therefore performed the direct test: joint residualization of four-category race (one-hot, White reference) together with its correlated cardiac comorbidities (heart failure and atrial fibrillation), followed by re-measurement of the race subgroup gap (Table S15). Across the six clean models, residualizing race alone left the gap essentially unchanged (0.071→0.070; +0.8%), residualizing the cardiac comorbidities slightly widened it (0.071→0.079; −11.3%, because removing finding-predictive cardiac signal degrades the classifier unevenly across groups), and the joint condition matched cardiac-only (0.079; −11.1%). No linear residualization of race or its clinical correlates narrowed the race subgroup gap, confirming that the gap is not an explicit, linearly-removable demographic or clinical-confounder pathway and is instead consistent with group-wise differences in finding-feature distributions (Section 4.1).

A limited external check on CheXpert Plus [28] reproduced this demographic decoupling qualitatively (near-zero race residualization drop alongside a persistent race subgroup gap; Table S16). This external check is *demographic-only*: because CheXpert Plus provides patient demographics but no ICD-linked comorbidity records, it cannot test whether the comorbidity OR→dependence hierarchy transfers across institutions. External validation of that relationship therefore remains untested and is the foremost open limitation of this work (Section 4.3), requiring an independent cohort with linked comorbidity records.

## 4. Discussion

### 4.1. A Three-Quantity Mechanism

The prevailing pipeline in medical-AI fairness research links encoding of a patient attribute to biased finding prediction to subgroup performance gaps, and motivates debiasing interventions at the encoding stage [1,2,3,7,29,30]. Our results separate three quantities on the same frozen embedding—encoding, residualization-sensitive dependence, and subgroup output gap—and show two dissociations between them. The first dissociation is between encoding and dependence: sex was encoded at AUROC 0.942 but contributed essentially zero under linear residualization, Black-vs-White race at AUROC 0.83 contributed only 0.0015 (rank 14 of 24), while the largest dependences came from clinical comorbidities that were less strongly encoded (heart failure 0.018, encoded at 0.774). The second dissociation is between dependence and subgroup output gap: on the same nine foundation models the four-category race subgroup AUROC gap averaged 0.069 while race residualization-sensitive dependence averaged 0.0015, a 30–75× scale difference. CheXzero, the driving example in Yang et al.’s recent vision-language foundation-model demographic-bias evaluation [10], showed a consistent pattern on our cohort—a descriptive within-cohort check—with race dependence 0.0013 alongside a race subgroup gap of 0.059 (maximum 0.122 for Atelectasis).

A parsimonious mechanism-level reading is that subgroup gaps in frozen-embedding plus linear-classifier architectures are driven primarily by finding-feature differences across demographic groups rather than by an explicit demographic-to-prediction pathway of the kind linear residualization targets. Two direct observations support this reading. First, jointly residualizing race and its correlated cardiac comorbidities does not narrow the race subgroup gap (Section 3.5, Table S15), so the gap is not reachable by linear removal of demographic or clinical-confounder directions. Second, positive finding base rates differ across race groups by up to 0.20 (Table S17, Panel A) while mean per-group AUROC is nearly equal (0.752–0.767), indicating that the four-category max–min gap reflects group-wise finding-feature and label-distribution differences—plus estimation variance in the smaller groups—rather than a systematic demographic performance deficit (per-cell AUROC covaries weakly and negatively with per-cell prevalence, ρ=−0.206, $p=1.3\times 10^{-3}$).

Whether reasoning-capable architectures (autoregressive multimodal LLMs that attend to demographic context in the decoder) reproduce or break this decoupling remains open.

Encoding measurements alone—which have informed much of the fairness literature [1,2,10]—are therefore upper bounds on the pathway-level contribution of an attribute, not direct estimates of it. This is precisely what separates age from sex and race. Age is both strongly encoded ($R^{2}=0.555$) *and* strongly associated with the thoracic findings (mean |log(OR)|=0.23; age-related cardiomegaly, pleural effusion, and pulmonary edema are directly visible on the radiograph), so a linear probe uses it (dependence 0.012). Sex and race are encoded as strongly or more so (AUROC 0.942 and 0.83) but are only weakly associated with these particular findings (sex mean |log(OR)|=0.09; race 0.24, concentrated in a few findings and largely carried by comorbidity correlates), so removing their linear signal barely changes prediction. In other words, what a model *can* read off the image (encoding) is dissociated from what *covaries with the finding label* (OR), and residualization-sensitive dependence tracks the latter. OR and dependence, in turn, are correlated downstream measurements rather than cause-and-effect: both reflect a shared radiological manifestation (attributes and findings that co-occur in reports also produce overlapping imaging features a frozen classifier exploits), and our regression quantifies how closely the two measurements track one another but does not identify OR as an upstream driver of dependence. Causal identification would require independent variation in exposure not available in a cross-sectional observational design.

Within our tested model set, pairwise rank correlations of attribute dependence exceeded 0.96 across all 15 model pairs (spanning a 20-fold effective-rank range and three training paradigms), and no model-level predictor added detectable variance beyond OR and finding intercepts (Table 4). The simplest reading is that the dependence hierarchy reflects data-level structure—which comorbidities and demographic attributes produce visible CXR changes that overlap with which finding labels—rather than idiosyncrasies of any one model, consistent with the FairMedFM [9] report of little architectural contribution. Effects below $\Delta R^{2}\approx 0.02$ would be undetectable with six models, so this is a bounded null rather than a claim of absolute architecture invariance.

### 4.2. Practical Implications for Fairness Auditing

For a target finding, the attributes most likely to change AUROC on linear residualization are those with the highest local odds ratio with the finding in the deployment cohort. The direction of this relationship generalized across 9 of 10 findings, but the slope did not (LOFO $R_{\mathrm{OOS}}^{2}$ was positive for 6 of 10 findings), so the OR-based hierarchy is best used as a starting template that is re-fitted to each deployment cohort. We validate this recommendation only for inpatient-skewed cohorts comparable to MIMIC-CXR: no public CXR cohort currently permits external testing of the comorbidity OR hierarchy (e.g., CheXpert Plus lacks ICD linkage; Section 4.3), and we do not claim it transfers unchanged to outpatient or screening populations. A fairness audit gains discriminating power by combining local odds ratios with encoding-based and protected-attribute analyses rather than relying on any single signal. Attribute identity itself contributed 16.2% of additional variance beyond OR and finding intercepts (specification M8), pointing to attribute-specific radiological visibility (for example, whether anemia produces a subtle or pronounced sign on a plain radiograph) as a natural extension of a purely OR-based prioritization scheme.

The findings for which a single consensus slope generalizes best (highest leave-one-finding-out $R_{\mathrm{OOS}}^{2}$; Table S2) are those dominated by a chronic, radiographically expressed comorbidity and spanning a wide dependence range: the cardiac and fluid-overload findings Edema ($R_{\mathrm{OOS}}^{2}=0.844$), Cardiomegaly (0.539), and Pleural Effusion (0.437)—each coupled to heart failure, the highest-dependence attribute—together with Pneumonia (0.713). Slope recovery fails (negative $R_{\mathrm{OOS}}^{2}$) for findings whose dependence range is compressed or whose label is unstable, even when the attribute rank direction is preserved: Atelectasis (the sole rank inversion, attributable to its 0.97 positive prevalence), Pneumothorax (an acute finding with a narrow dependence range; $R_{\mathrm{OOS}}^{2}=-0.997$ despite Spearman ρ=0.63), the nonspecific Lung Opacity, and Enlarged Cardiomediastinum (a CheXpert label hierarchically entangled with Cardiomegaly). Because $R_{\mathrm{OOS}}^{2}$ is scored against each finding’s own dependence variance, a finding with little finding-specific slope to recover yields a strongly negative value. This finding-level heterogeneity is the per-finding counterpart of the attribute-identity effect noted above: the same finding-specific radiological expression also limits how well a single consensus slope transfers across findings—reinforcing our recommendation to re-fit local odds ratios per deployment cohort rather than treat the hierarchy as a universal constant.

The per-attribute effects were individually modest—0.018 AUROC for the strongest attribute, compared with the 0.05–0.15 subgroup gaps reported in prior fairness audits [7,9]—yet systematic across all 1440 observations, and residualizing the top three jointly produced a combined drop of 0.026. These effect sizes are deliberately small and should be read as an *audit-level prioritization signal, not a bedside-level quantity*: a 0.018 AUROC dependence does not by itself change an individual diagnostic decision, but it consistently flags which attribute-finding pairs warrant fairness scrutiny. The hierarchy is metric-robust—recomputing dependence with the Matthews correlation coefficient [31] reproduces the identical attribute ranking (Spearman ρ=1.00 with the AUROC ranking; heart failure highest, sex and race ≈0; Table S20)—so the small magnitudes reflect a genuine, metric-invariant property of the data rather than an artifact of AUROC. Because top-ranked attributes share correlated embedding directions (heart failure × atrial fibrillation tetrachoric ϕ=0.44), individual drops are not additive: simultaneous residualization of the top three yielded approximately 44% less joint drop than the sum of the three individual drops (Table S9). The practical recommendation is diagnostic rather than prescriptive: OR indicates which attribute-finding pairs to evaluate first, not where to intervene.

### 4.3. Limitations and Future Directions

**Cohort generalizability.** OR and attribute dependence were computed on the same MIMIC-CXR cohort, so within-cohort shared structure cannot be fully separated from a genuine OR-dependence relationship. Random patient-level splitting within a single 2011–2016 window does not test temporal acquisition-protocol drift, and the ICU-heavy Beth Israel cohort (60.5% portable anteroposterior) is not representative of general radiology; a prospective temporal hold-out within MIMIC or evaluation on a post-2020 CXR cohort would strengthen external validity. Within MIMIC, leave-one-attribute-out and leave-one-finding-out cross-validation provide held-out-unit robustness ($R_{\mathrm{OOS}}^{2}=0.459$), and a limited external check on CheXpert Plus reproduced the demographic encoding–dependence and dependence–subgroup-gap dissociations (Section 3.5, Table S16). The central *OR → dependence* relationship, however, cannot be externally validated there because CheXpert Plus lacks ICD linkage, and no public CXR cohort currently links comorbidity EHR (the VA-CXR cohort used in recent domain-shift work [32] is restricted to VA users). The comorbidity attribute ranking we report is therefore best treated as a cohort-specific starting template, to be re-fitted with local ORs and validated against ICD-linked external data, rather than a universal hierarchy.

**Fine-tuning regime.** Our analysis applied to *frozen* foundation-model embeddings, while deployed systems are commonly fine-tuned. A single LoRA experiment on RAD-DINO collapsed normalized effective rank from 0.514 to 0.152 after adaptation (Table S5). One model and one adaptation method are not enough to establish whether the encoding-dependence dissociation persists, weakens, or strengthens under fine-tuning; we flag the question rather than answer it.

**Linearity of the dependence measure.** Attribute dependence is defined by *linear* (ridge) residualization, which removes the linear component of an attribute from the embedding (for a binary attribute, the group-mean difference). For the linear finding probe we use, this removes the attribute’s linearly-usable signal; the dependence hierarchy is moreover robust to nonlinear residualization of the continuous attributes (Spearman ρ=1.00; Table S18) and to nonlinear concept erasure in a kernel feature space, where the ranking is unchanged and sex and race remain the two lowest attributes (Table S19). A decodability probe shows, however, that demographics remain recoverable after linear residualization (sex/race AUROC 0.97/0.91), and the kernel-space erasure leaves a small non-zero demographic residual: distributed nonlinear demographic structure is *not* fully removed, so a nonlinear classifier (or a fine-tuned head) could in principle access it. We therefore interpret dependence as a measure of *linear* use, matching the linear/affine debiasing methods our results bear on; stronger optimized nonlinear erasure or adversarial debiasing, evaluated by its ability to reduce subgroup gaps, is the appropriate tool for an unconditional mitigation claim and remains future work.

**Measurement caveats.** Five data-level caveats attenuate but do not reverse our findings.

**Labeler noise.** Rule-based NLP labels from the CheXpert labeler may correlate errors with patient complexity; its internal validation against radiologist readings reported F1 scores of 0.70–0.95 across the 14 CheXpert labels [14], placing our labels in a well-characterized noise range. We use the U-Ignore policy (uncertain mentions masked); recomputing all attribute-finding odds ratios under the alternative U-Zero policy (uncertain treated as negative) preserved the attribute ranking (Spearman ρ=0.94, Pearson r=0.95), so the uncertainty-label convention does not drive the OR → dependence relationship.

**ICD under-coding.** ICD-based comorbidities undercount conditions with low coding sensitivity, particularly obesity and depression.

**Image-level weighting.** The image-level unit over-weights frequently imaged patients; the IPW and patient-level OR sensitivities reported in Section 3.2 preserve the attribute ranking (ρ=0.963; Table S9), but more sophisticated patient-weighting schemes are not explored here.

**ICD timing.** Our patient-level ever-coded ICD definition (Section 2.1) does not distinguish codes assigned before versus after the index CXR, so a fraction of comorbidity labels reflect diagnoses confirmed at discharge; this creates a weak label-feature circularity for imaging-adjudicable comorbidities (a CXR read as suggestive of heart failure can contribute to the eventual heart-failure ICD) that would inflate attribute-finding ORs for such conditions. To bound this concern we stratified the 24 attributes into 5 imaging-adjudicable conditions (heart failure, respiratory failure, pulmonary fibrosis, COPD, cancer history) and 19 non-adjudicable attributes (the 4 demographics plus 15 comorbidities diagnosed by blood test, ECG, BP measurement, BMI, psychiatric assessment, brain imaging, or clinical history) and re-fit the OR → dependence regression on each subset (Table S14). The slope on the non-adjudicable subset (β=0.027, $R^{2}=0.512$, n=1140) was essentially identical to the full-data slope (β=0.029, $R^{2}=0.506$), and the slope-equality test between the two subsets was not rejected under attribute-clustered robust standard errors (p=0.32); timing-driven circularity is therefore not a plausible driver of the main OR → dependence finding. The main encoding-dependence dissociation is separately insensitive to this concern because sex, BMI, and race – which are not ICD-derived – show the same pattern. Each of these biases shrinks the OR-dependence relationship toward the null, so the reported covariation is a lower bound on the true relationship.

**Residual confounding.** Attribute-finding odds ratios may also be shaped by acquisition practice and care-process variables. Adjusting for view position by Mantel-Haenszel stratification (AP versus PA) left the odds ratios essentially unchanged (cell-level Spearman ρ=0.96 with the crude OR; mean |Δlog(OR)|=0.04), so acquisition view is not a material confounder. Treatment history and other care-process variables are not available in the linked tables and cannot be adjusted for; we name this as residual unmeasured confounding. This is consistent with our framing that OR and dependence are correlated downstream measurements rather than a causal relationship (Section 4.1), and multicollinearity among the modelled comorbidities was modest (all VIF <2; Section 2.4).

**Mitigation strategies beyond linear residualization.** Because linear demographic residualization does not detectably reduce the race subgroup gap beyond ∼3% in our test (MDES-bounded; Section 3.5) and the residual gap tracks group-wise finding base rates and feature distributions rather than a removable demographic direction (Section 4.1, Table S17), interventions confined to stripping a linear attribute direction are unlikely to suffice. The diagnosis points to four classes of alternative, matched to the mechanism we observe: (i) *data balancing and reweighting* and (ii) *distribution-matching domain adaptation*, which target the across-group base-rate and feature-distribution differences directly rather than the representation; (iii) *fairness-aware adversarial training* and *causal/invariant representation learning*, which can remove the nonlinear attribute information that linear residualization leaves intact (an MLP still decodes sex and race after linear residualization; Table S18); and (iv) group-specific operating-point calibration for threshold-level fairness. We frame these as the strategies our findings motivate rather than ones we evaluate here, and note that their relative benefit is itself an open empirical question on cohorts with linked EHR.

**Priority next steps.** Three follow-ups extend the design rather than retrofit it. (i) *Per-attribute residualization as a fairness-pathway test.* The top-three joint residualization we report (Section 3.4) produced a roughly uniform AUROC drop across cells (∼0.026), leaving insufficient between-cell variance to map residualization-induced drops onto subgroup-gap changes. Per-attribute drops span two orders of magnitude across the 24 attributes and would enable a more discriminating test on the present cohort, using the regression framework already in place. (ii) *Reaching the race subgroup gap.* We performed the direct joint race-plus-cardiac-comorbidity residualization (Section 3.5, Table S15): residualizing four-category race together with heart failure and atrial fibrillation does not narrow the race subgroup gap (and slightly widens it), so the gap is not a linearly-removable demographic or clinical-confounder pathway in the frozen-embedding regime. What remains open is whether nonlinear concept-erasure or causal-intervention methods, or directly modeling the group-wise finding-feature distribution differences that Major-finding base rates reveal (Section 4.1), can reduce it. Yang et al.’s recent finding that vision-language foundation models encode demographics more strongly than board-certified radiologists [10] makes closing this gap by some non-linear-residualization route particularly consequential. (iii) *Fine-tuning regime extension.* Testing the dissociation across full fine-tuning, LoRA, and adapter-only regimes on multiple models is the direct follow-up to the fine-tuning limitation above and the most informative extension for deployed-system relevance.

## 5. Conclusions

Across nine CXR foundation models on MIMIC-CXR, three fairness-relevant quantities measured on the same frozen embedding—attribute encoding, residualization-sensitive dependence, and subgroup AUROC gap—dissociated along two axes. Encoding did not covary with dependence: sex (AUROC 0.942, mean dependence <0.001) and Black-vs-White race (AUROC 0.71–0.90, mean dependence 0.0015) were strongly encoded but weakly used by the linear probe, while clinical comorbidities were the attributes that actually moved finding prediction when residualized. Dependence did not covary with subgroup gap: race subgroup AUROC gaps were 30–75× larger than race residualization drops, and CheXzero—the driving example in the recent demographic-bias evaluation of Yang et al. [10]—showed a consistent pattern on our cohort (race dependence 0.0013, race subgroup gap 0.059). Attribute-finding odds ratios accounted for approximately half of dependence variance, and model architecture and training objective added no detectable variance beyond OR. In the specific top-three joint residualization we tested, AUROC fell by 0.026 without detectably narrowing sex or age subgroup gaps (MDES = 0.0019)—a pattern more consistent with clinical confounding than with a direct demographic-bias pathway for that procedure.

For practitioners deploying CXR foundation models as frozen embedding backbones on inpatient-skewed cohorts comparable to MIMIC-CXR, our results support a two-step pre-deployment fairness check: (i) compute local odds ratios between the target finding and high-prevalence comorbidities (heart failure, atrial fibrillation, age tertiles) in the deployment cohort; (ii) prioritize debiasing evaluation for attributes with the highest local OR, rather than for those that are most strongly encoded. Linearly residualizing a demographic signal from the embedding did not detectably narrow the subgroup gap beyond ∼0.002 in our test (MDES bound vs. observed race gap 0.069), so this intervention alone does not substantially narrow the gap. Joint residualization of race with its correlated cardiac comorbidities did not narrow the race subgroup gap (Section 3.5), and the hierarchy was preserved under nonlinear concept erasure (Section 2.4); robustness under fine-tuning and cross-institutional transfer of the attribute ranking remain the principal unresolved questions.

## Figures and Tables

**Table 1:** Cohort descriptive statistics for the MIMIC-CXR study population (*n* = 230,697 frontal chest radiographs from 60,518 patients). Age, sex, and BMI are shown at both patient and image level; comorbidity prevalence is derived from ICD-9/10 codes linked to the hospital admission associated with each image. Image-level prevalence exceeds patient-level prevalence because patients with more comorbidities undergo more imaging studies.

**Panel A. Demographics**		
**Characteristic**	**Patient-Level (n = 60,518)**	**Image-Level (n = 230,697)**
Age (years), mean ± SD (median, IQR)	56.9 ± 19.5 (58.0, 43.0–72.0)	60.8 ± 17.5 (62.0, 50.0–74.0)
Sex, female/male	31,607 (52.2%)/ 28,911 (47.8%)	106,482 (46.2%)/ 124,215 (53.8%)
BMI (kg/m^2^), mean ± SD (available %)	28.5 ± 7.1 (70.4%)	28.1 ± 7.5 (78.7%)
Images per patient, mean	—	3.81
**Panel B. Comorbidity prevalence (ICD-derived)**		
**Comorbidity**	**Patient-Level n (%)**	**Image-Level n (%)**
Hypertension	25,298 (41.8%)	119,248 (51.7%)
Hyperlipidemia	23,262 (38.4%)	115,234 (50.0%)
Anemia	17,353 (28.7%)	112,987 (49.0%)
Acute kidney injury	13,790 (22.8%)	98,987 (42.9%)
Heart failure	10,769 (17.8%)	78,280 (33.9%)
Diabetes	13,885 (22.9%)	75,744 (32.8%)
Coronary artery disease	12,324 (20.4%)	73,002 (31.6%)
Cancer history	12,020 (19.9%)	70,237 (30.4%)
Atrial fibrillation	10,561 (17.5%)	70,230 (30.4%)
Chronic kidney disease	10,147 (16.8%)	68,523 (29.7%)
Depression	12,178 (20.1%)	67,460 (29.2%)
COPD	6517 (10.8%)	47,538 (20.6%)
Obesity	7890 (13.0%)	42,893 (18.6%)
Hypothyroidism	7157 (11.8%)	39,064 (16.9%)
Respiratory failure	4304 (7.1%)	39,054 (16.9%)
Asthma	6952 (11.5%)	37,402 (16.2%)
Liver disease	3234 (5.3%)	22,980 (10.0%)
Smoking history	3643 (6.0%)	19,352 (8.4%)
Stroke	2298 (3.8%)	14,667 (6.4%)
Pulmonary fibrosis	547 (0.9%)	4779 (2.1%)

**Table 2:** Foundation models used in the main analysis.

Model	Architecture	Training Paradigm	Input Size	Embedding Dim
ResNet50-ImageNet [17]	CNN (ResNet50)	Supervised (ImageNet)	224	2048
DINOv2-base [18]	ViT-B/14	Self-supervised (142M images)	518	768
BiomedCLIP [19]	ViT-B/16	Vision-language (15M pairs)	224	512
XRV-DenseNet-nih [20]	CNN (DenseNet121)	Supervised (NIH CXR14)	224	1024
CLIP-ViT-B16 [21]	ViT-B/16	Vision-language (LAION-2B)	224	512
ConvNeXtV2-Base [22]	ConvNeXtV2-Base	Self-supervised (ImageNet-22k)	224	1024

**Table 3:** Top 5 (ranks 1–5) of 24 attributes by mean attribute dependence, the protected demographic *race* at its mid-table rank, and bottom 5 (ranks 20–24); mean across 6 models × 10 findings (full ranking, Table S1).

Rank	Attribute	Type	Encoding	Mean |log(OR)|	Mean AUROC Drop	95% CI
1	Heart failure	Comorbidity	0.774	0.383	0.018	[0.013, 0.023]
2	Atrial fibrillation	Comorbidity	0.775	0.347	0.015	[0.012, 0.018]
3	Age	Demographic	$R^{2}=0.555$	0.226	0.012	[0.009, 0.015]
4	Acute kidney injury	Comorbidity	0.708	0.354	0.011	[0.010, 0.012]
5	Anemia	Comorbidity	0.672	0.282	0.009	[0.008, 0.010]
14	Race (Black-vs-White)	Demographic	0.830	0.237	0.0015	[0.0010, 0.0020]
20	Liver disease	Comorbidity	0.706	0.114	<0.001	[−0.000, 0.001]
21	Sex	Demographic	0.942	0.091	<0.001	[−0.000, 0.001]
22	Asthma	Comorbidity	0.636	0.080	<0.001	[−0.000, 0.000]
23	Depression	Comorbidity	0.588	0.081	<0.001	[−0.000, 0.000]
24	Smoking history	Comorbidity	0.649	0.109	<0.001	[−0.000, 0.000]

**Table 4:** Nested regression: predictors of attribute dependence (n=1440, 24 attributes × 10 findings × 6 models). ^†^ M1a estimated on binary-attribute subset (n=1320) where encoding metric is unified. Full specifications, including cluster-robust and mixed-effects corrections, appear in Table S6.

Model	Formula	$R^{2}$	$\Delta R^{2}$	F-Test *p*
M1	drop ∼|log(OR)|	0.506	—	—
M1a	drop ∼|log(OR)|+ encoding ^†^	0.555 ^†^	0.006 ^†^	<0.001 ^†^
M2	drop ∼|log(OR)|+log(erank)	0.506	0.000	0.678
M5	drop ∼|log(OR)|+ finding	0.615	0.109	—
M6a	drop ∼|log(OR)|+ finding +log(erank)	0.615	0.000	0.665
M6b	drop ∼|log(OR)|+ finding + model identity	0.615	0.000	0.907
M7a	drop ∼|log(OR)|×log(erank)+ finding	0.615	0.000	0.803
M7b	drop ∼|log(OR)|× model + finding	0.616	0.001	0.905
M8	drop ∼|log(OR)|+ finding + attribute	0.777	0.162	—

## Data Availability

MIMIC-CXR v2.0 [12,13] and MIMIC-IV [15,16] are publicly available through PhysioNet [34] (https://physionet.org, accessed on 10 February 2026) under credentialed data use agreements. The analysis code (residualization, nested-regression, race-probing, and biostatistical-diagnostics scripts) and the aggregate result tables underlying all tables and figures (result CSVs cited in Tables S1–S21) are publicly available at https://github.com/honeia85/cxr-attribute-dependence, archived at Zenodo (https://doi.org/10.5281/zenodo.21142236). Extracted foundation-model embeddings are derived from the credentialed MIMIC-CXR and CheXpert datasets and cannot be redistributed under the PhysioNet and Stanford AIMI data use agreements; they can be regenerated from the released extraction scripts using credentialed data, or obtained by credentialed users on request to the corresponding author.

## Supplementary Materials

The following supporting information can be downloaded at: https://www.mdpi.com/article/10.3390/diagnostics16132030/s1, Table S1: Full Attribute Dependence Hierarchy (24 Attributes × 6 Models); Table S2: Leave-One-Finding-Out Cross-Validation Results; Table S3: Fairness Gap Analysis by Model; Table S4: Odds Ratios: 24 Patient Attributes × 10 Clinical Findings; Table S5: LoRA Fine-Tuning Effects on Embedding Geometry (RAD-DINO); Table S6: Full Nested Regression Models with Robustness Checks; Table S7: Patient-Level vs. Image-Level Odds Ratio Sensitivity Analysis; Table S8: Baseline Linear Probe AUROC (6 Clean Models × 10 Clinical Findings); Table S9: Robustness Checks: Inverse-Probability Weighting and Simultaneous Residualization; Table S10: Pairwise Rank Correlations of Attribute Dependence; Table S11: Residual Diagnostics and Robust-Regression Sensitivity for M1; Table S12: Nine-Model Sensitivity Analysis of the Nested Regression; Table S13: Race as the 24th Attribute: Residualization, Odds Ratios, and Subgroup Gaps; Table S14: ICD Timing Sensitivity: Stratified OR → Dependence Regression by Adjudicability; Table S15: Direct Race-Gap Test: Joint Residualization of Race and Cardiac Comorbidities; Table S16: CheXpert Plus External Sanity Check (Demographic Components); Table S17: Sources of the Race Subgroup Gap: Per-Group Base Rates and Calibration (MIMIC-CXR); Table S18: Nonlinearity Check: MLP Residualization, MLP Use, and Demographic Decodability; Table S19: Nonlinear Concept Erasure with a Random-Direction Control; Table S20: Dependence Hierarchy under the Matthews Correlation Coefficient (MCC); Table S21: Multi-Metric Subgroup Fairness with Bootstrap Confidence Intervals; Figure S1: Per-Model OR → Dependence Regression Slopes; Figure S2: Within-Finding OR → Dependence Scatter are provided in the separate Supplementary Materials file, together with the Supplementary Methods, Checklist for Artificial Intelligence in Medical Imaging (CLAIM) 2024 [33], and STROBE checklists.

## Abbreviations

The following abbreviations are used in this manuscript:

AUROC	Area Under the Receiver Operating Characteristic Curve
BMI	Body Mass Index
CI	Confidence Interval
CLAIM	Checklist for Artificial Intelligence in Medical Imaging
CNN	Convolutional Neural Network
COPD	Chronic Obstructive Pulmonary Disease
CXR	Chest X-Ray
EHR	Electronic Health Record
erank	Effective Rank
FDR	False Discovery Rate
ICD	International Classification of Diseases
IRB	Institutional Review Board
LOAO	Leave-One-Attribute-Out
LOFO	Leave-One-Finding-Out
LoRA	Low-Rank Adaptation
MDES	Minimum Detectable Effect Size
MLP	Multilayer Perceptron
OLS	Ordinary Least Squares
OR	Odds Ratio
STROBE	Strengthening the Reporting of Observational Studies in Epidemiology
VIF	Variance Inflation Factor
ViT	Vision Transformer
