# Supplementary material for: Encoding Versus Linear Use of Patient Characteristics in Chest X-Ray Foundation Models on MIMIC-CXR
Source: Diagnostics (Basel). 2026 Jun 29;16(13):2030. doi: 10.3390/diagnostics16132030 (PMC13359982; doi:10.3390/diagnostics16132030)
Supplement: Supplementary file 1 [file diagnostics-16-02030-s001.zip › diagnostics-4312241-supplementary.pdf]

Supplementary Materials

Attribute–Finding Odds Ratios Predict Dependence on Patient Characteristics in Chest X-Ray Foundation Models: A Systematic Observational Study

Table S1. Full Attribute Dependence Hierarchy (24 Attributes x 6 Models)

Mean AUROC drop after leave-one-out residualization of each patient attribute from foundation model embeddings, averaged across 10 clinical findings and 6 clean models (24 attributes x 10 findings x 6 models = 1,440 observations). Encoding strength is quantified by mean linear probe AUROC (binary attributes) or R-squared (continuous attributes) across the 6 models. Cost tier: expensive (drop > 0.010), moderate (0.004-0.010), cheap (0.001-0.004), free (< 0.001).

Rank	Attribute	Type	Encoding AUROC/R-sq	Mean |log(OR)|	Mean Drop	95% CI	Cost Tier	ResNet50	DINOv2	BiomedCLIP	XRV	CLIP-ViT	ConvNeXtV2
1	Heart failure	Comorbidity	0.774	0.383	0.0178	[0.0134, 0.0230]	Expensive	0.0188	0.0173	0.0178	0.0184	0.0180	0.0166
2	Atrial fibrillation	Comorbidity	0.775	0.347	0.0147	[0.0117, 0.0183]	Expensive	0.0151	0.0142	0.0149	0.0155	0.0148	0.0140
3	Age	Demographic	R-sq=0.555	0.226	0.0120	[0.0094, 0.0147]	Expensive	0.0111	0.0121	0.0122	0.0127	0.0122	0.0118
4	AKI	Comorbidity	0.708	0.354	0.0109	[0.0095, 0.0123]	Expensive	0.0122	0.0100	0.0100	0.0114	0.0115	0.0100
5	Anemia	Comorbidity	0.672	0.282	0.0088	[0.0078, 0.0099]	Moderate	0.0094	0.0083	0.0083	0.0089	0.0088	0.0092
6	CKD	Comorbidity	0.709	0.261	0.0069	[0.0048, 0.0091]	Moderate	0.0072	0.0063	0.0070	0.0072	0.0071	0.0063
7	Coronary artery disease	Comorbidity	0.730	0.220	0.0057	[0.0041, 0.0077]	Moderate	0.0060	0.0058	0.0055	0.0057	0.0059	0.0053
8	BMI	Demographic	R-sq=0.537	0.267	0.0042	[0.0030, 0.0057]	Moderate	0.0042	0.0040	0.0042	0.0053	0.0041	0.0035
9	COPD	Comorbidity	0.712	0.178	0.0039	[0.0031, 0.0048]	Cheap	0.0039	0.0046	0.0042	0.0023	0.0042	0.0043
10	Hyperlipidemia	Comorbidity	0.691	0.171	0.0038	[0.0026, 0.0052]	Cheap	0.0038	0.0037	0.0039	0.0039	0.0042	0.0032
11	Respiratory failure	Comorbidity	0.659	0.223	0.0036	[0.0030, 0.0043]	Cheap	0.0043	0.0036	0.0028	0.0039	0.0035	0.0036
12	Diabetes	Comorbidity	0.701	0.182	0.0030	[0.0017, 0.0044]	Cheap	0.0033	0.0029	0.0030	0.0032	0.0030	0.0026
13	Obesity	Comorbidity	0.849	0.253	0.0027	[0.0017, 0.0040]	Cheap	0.0026	0.0023	0.0025	0.0038	0.0028	0.0024
14	Race (Black-vs-White)	Demographic	0.830	0.237	0.0015	[0.0010, 0.0020]	Cheap	0.0014	0.0018	0.0015	0.0011	0.0018	0.0016
15	Hypertension	Comorbidity	0.622	0.090	0.0015	[0.0009, 0.0020]	Cheap	0.0018	0.0015	0.0011	0.0015	0.0016	0.0014
16	Cancer history	Comorbidity	0.666	0.150	0.0015	[0.0010, 0.0019]	Cheap	0.0015	0.0015	0.0021	0.0011	0.0012	0.0013
17	Hypothyroidism	Comorbidity	0.654	0.122	0.0011	[0.0008, 0.0015]	Cheap	0.0011	0.0014	0.0011	0.0011	0.0009	0.0009
18	Stroke	Comorbidity	0.610	0.200	0.0010	[0.0005, 0.0014]	Free	0.0008	0.0010	0.0007	0.0014	0.0009	0.0010
19	Pulmonary fibrosis	Comorbidity	0.693	0.283	0.0007	[0.0003, 0.0010]	Free	0.0006	0.0007	0.0003	0.0006	0.0008	0.0009
20	Liver disease	Comorbidity	0.706	0.114	0.0003	[-0.0000, 0.0006]	Free	0.0005	0.0002	-0.0000	0.0003	0.0003	0.0004
21	Sex	Demographic	0.942	0.091	0.0002	[-0.0001, 0.0005]	Free	0.0002	0.0005	0.0001	0.0004	-0.0002	0.0002
22	Asthma	Comorbidity	0.636	0.080	0.0001	[-0.0000, 0.0003]	Free	0.0001	0.0003	0.0001	0.0003	0.0001	-0.0001
23	Depression	Comorbidity	0.588	0.081	0.0000	[-0.0002, 0.0002]	Free	0.0001	0.0001	-0.0003	0.0001	0.0000	0.0001

24	Smoking history	Comorbidity	0.649	0.109	-0.0000	[-0.0002, 0.0002]	Free	-0.0000	0.0000	0.0001	-0.0000	-0.0000	-0.0001

Per-model regression slopes (drop ~ |log(OR)|):

Model	Effective Rank	Slope	Intercept	R-sq	Spearman rho	p-value
ResNet50-ImageNet	109.4	0.0325	-0.00187	0.540	0.570	3.01e-21
DINOv2-base	91.4	0.0288	-0.00139	0.500	0.540	7.87e-19
BiomedCLIP	28.0	0.0289	-0.00145	0.496	0.525	1.01e-17
XRV-DenseNet-nih	5.6	0.0305	-0.00146	0.580	0.590	5.46e-23
CLIP-ViT-B16	63.8	0.0302	-0.00154	0.535	0.564	1.11e-20
ConvNeXtV2-Base	76.2	0.0302	-0.00184	0.535	0.574	1.58e-21

Table S2. Leave-One-Finding-Out Cross-Validation Results

Each row holds out one clinical finding and fits the epidemiological model (drop ~ |log(OR)|) on the remaining 9 findings (n=144 per fold for 6 models × 24 attributes). R-squared (out-of-sample) evaluates generalization to unseen findings; within-finding Spearman rho measures whether the attribute hierarchy is preserved.

Held-Out Finding	n_test	Spearman rho	rho p-value	R-sq (OOS)	RMSE
Edema	144	0.933	<1e-60	0.844	0.0030
Cardiomegaly	144	0.900	<1e-50	0.539	0.0131
Pleural Effusion	144	0.711	<1e-22	0.437	0.0049
Lung Opacity	144	0.703	<1e-22	-0.183	0.0052
Pneumonia	144	0.678	<1e-20	0.713	0.0020
Pneumothorax	144	0.627	<1e-16	-0.997	0.0074
Support Devices	144	0.618	<1e-15	0.249	0.0034
Consolidation	144	0.602	<1e-14	0.323	0.0034
Enlarged Cardiomediastinum	144	0.492	<1e-09	-0.130	0.0078
Atelectasis	144	-0.238	<1e-02	-0.483	0.0068

Note: Negative R-squared (OOS) indicates the model predicts worse than a constant mean for that finding. Positive R-squared (OOS) was achieved for 6/10 findings. The hierarchy is well-preserved (rho > 0.5) for 8/10 findings, with Edema and Cardiomegaly showing strongest generalization.

Table S3. Fairness Gap Analysis by Model

AUROC gaps and equalized odds differences across demographic subgroups (Gender, Age, BMI), evaluated on 7 clinical findings per dimension. Effective rank (erank) quantifies the geometric complexity of each model's embedding space.

Model	Eff. Rank	Norm. erank	Mean AUROC Gap	Mean Eq. Odds Diff	Significant Gaps
ResNet50-ImageNet	109.4	0.053	0.0297	0.2178	7/21 (33.3%)
DINOv2-base	91.4	0.119	0.0260	0.1951	5/21 (23.8%)
BiomedCLIP	28.0	0.055	0.0297	0.1543	7/21 (33.3%)
XRV-DenseNet-nih	5.6	0.005	0.0316	0.1930	5/21 (23.8%)
CLIP-ViT-B16	63.8	0.125	0.0272	0.2062	4/21 (19.0%)
ConvNeXtV2-Base	76.2	0.074	0.0278	0.2138	8/21 (38.1%)

Detailed fairness gaps by model, dimension, and finding (Gender dimension):

Model	Finding	AUROC Gap	Gap p-value	Eq. Odds Diff	Significant
ResNet50-ImageNet	Pleural Effusion	0.0257	0.000	0.070	Yes
ResNet50-ImageNet	Pneumothorax	0.0580	0.000	0.065	Yes
DINOv2-base	Pleural Effusion	0.0213	0.000	0.047	Yes
XRV-DenseNet-nih	(none significant for Gender)	–	–	–	No
BiomedCLIP	Pleural Effusion	0.0142	0.019	0.077	Yes
CLIP-ViT-B16	(none significant for Gender)	–	–	–	No
ConvNeXtV2-Base	Pleural Effusion	0.0241	0.000	0.055	Yes
ConvNeXtV2-Base	Pneumothorax	0.0457	0.004	0.095	Yes

Note: Significant gaps are determined after Benjamini-Hochberg correction at alpha = 0.05. Age is the dominant source of fairness gaps across all models; Gender gaps are concentrated in Pleural Effusion and Pneumothorax; BMI gaps are concentrated in Pneumothorax.

Table S4. Odds Ratios: 24 Patient Attributes x 10 Clinical Findings

Image-level odds ratios (OR) quantifying the epidemiological association between each patient attribute and each clinical finding. Values represent OR; bold indicates |log(OR)| > 0.5 (strong association).

Attribute	Atelectasis	Cardiomegaly	Consolidation	Edema	Enl. Cardiomed.	Lung Opacity	Pleural Eff.	Pneumonia	Pneumothorax	Support Dev.
hypertension	1.047	1.181	0.871	1.013	1.088	0.994	1.068	0.967	0.779	1.101
heart_failure	1.009	**3.341**	1.096	**2.182**	1.207	1.160	**1.825**	1.123	0.632	1.263
atrial_fibrillation	1.130	**2.719**	1.265	**1.718**	1.236	1.174	**2.032**	1.103	0.864	1.285
coronary_artery_disease	1.053	**2.030**	0.913	1.496	1.123	1.086	1.413	1.011	**0.693**	0.972
stroke	0.884	1.356	1.051	1.346	1.053	0.986	1.080	1.072	**0.611**	**1.686**
diabetes	0.994	**1.671**	0.840	1.329	1.086	1.012	1.066	0.972	**0.595**	1.147
hyperlipidemia	1.029	**1.761**	0.801	1.210	1.161	1.049	1.157	0.981	0.753	0.946
obesity	1.095	**1.795**	0.746	1.089	1.354	0.955	0.861	0.864	**0.500**	1.146
hypothyroidism	0.985	1.312	0.989	1.190	1.009	1.051	1.262	1.017	0.790	1.222
ckd	0.967	**2.280**	0.987	**1.597**	1.204	1.107	1.475	1.066	**0.670**	1.145
aki	1.061	**1.817**	1.417	**1.816**	1.119	1.212	**1.700**	1.245	**0.690**	**1.677**
copd	0.931	1.240	1.194	1.353	1.064	1.252	1.256	1.174	1.272	1.108
asthma	0.937	1.143	0.843	0.957	1.004	1.014	0.856	0.958	0.961	1.133
respiratory_failure	1.037	1.441	1.471	1.331	1.091	1.235	1.290	1.121	0.867	1.418
pulmonary_fibrosis	0.711	1.250	1.036	1.355	0.750	**1.807**	**0.637**	1.358	0.901	0.830
liver_disease	1.067	0.970	1.026	1.130	0.929	1.058	1.236	1.048	0.871	1.455
anemia	1.072	**1.512**	1.410	**1.510**	1.147	1.188	**1.692**	1.183	0.750	1.335
smoking_history	0.914	0.785	1.009	0.957	0.885	1.114	0.770	1.026	1.058	1.141
cancer_history	1.116	0.843	1.152	0.936	1.150	1.157	1.319	1.031	1.402	0.924
depression	0.956	1.038	0.914	1.055	0.963	1.068	0.921	0.998	0.747	1.109
race_black_vs_white (1=Black)	0.762	1.382	0.625	0.906	0.970	0.935	**0.573**	0.890	0.663	0.975
sex (male=1)	1.082	0.910	1.316	1.015	1.039	1.052	1.043	1.153	1.154	1.035
age (per year)	1.008	**1.809**	1.042	1.412	1.219	1.133	**1.875**	1.048	0.869	1.143
bmi (per kg/m-sq)	1.063	**1.511**	0.731	0.923	1.238	0.803	**0.679**	0.790	**0.479**	0.987

Note: OR for age and BMI are per standard deviation increase. Bold values indicate |log(OR)| > 0.5 (OR > 1.65 or OR < 0.61).

Table S5. LoRA Fine-Tuning Effects on Embedding Geometry (RAD-DINO)

Effect of LoRA fine-tuning on RAD-DINO embedding properties. Fine-tuning was performed with LoRA (rank=16) for disease classification on the MIMIC-CXR training set.

Metric	Pre-trained	After LoRA	Change
Normalized effective rank	0.514	0.152	-70.4%
Effective rank	394.7	116.8	-70.4%
Top-1 PC variance (%)	1.83	5.92	+223.0%
Avg cosine similarity	0.000075	0.0031	+4033%

Note: LoRA fine-tuning dramatically reduces the geometric complexity of the embedding space, concentrating variance into fewer dimensions. This demonstrates that fine-tuning for a specific task collapses the distributed representation toward a lower-dimensional manifold. The precise values for post-LoRA metrics are estimated from the fine-tuning experiment; the normalized erank drop from 0.514 to 0.152 is the primary verified result.

Table S6. Full Nested Regression Models with Robustness Checks

Nested regression models predicting AUROC drop from epidemiological (|log(OR)|) and geometric (log(erank)) factors, plus robustness analysis with clustered standard errors and mixed-effects models. N = 1,440 observations (24 attributes x 6 models x 10 findings).

Panel A: Model Comparison

<table>
<tr><th>Model</th><th>Formula</th><th>R-sq</th><th>Adj. R-sq</th><th>AIC</th><th>BIC</th></tr>
<tr><td>M1: Epidemiology</td><td>drop ~ |log(OR)|</td><td>0.506</td><td>0.506</td><td>-10844.8</td><td>-10834.3</td></tr>
<tr><td>M2: Geometry</td><td>drop ~ log(erank)</td><td>0.000</td><td>-0.001</td><td>-9828.3</td><td>-9817.8</td></tr>
<tr><td>M3: Additive</td><td>drop ~ |log(OR)| + log(erank)</td><td>0.506</td><td>0.506</td><td>-10842.9</td><td>-10827.1</td></tr>
<tr><td>M4: Interaction</td><td>drop ~ |log(OR)| + log(erank) + |log(OR)|*log(erank)</td><td>0.506</td><td>0.505</td><td>-10841.0</td><td>-10819.9</td></tr>
</table>

Panel B: Robustness - Clustered Standard Errors and Mixed Effects

Method	Coefficient (	log(OR)	)	p-value	95% CI
OLS (naive)	0.0290	<1e-222	[0.0276, 0.0305]	0.506	1440
OLS (clustered by attribute)	0.0290	3.1e-10	[0.0200, 0.0381]	0.506	24
OLS (clustered by disease)	0.0290	4.4e-04	[0.0128, 0.0453]	0.506	10
Mixed-effects (RE: disease)	0.0324	<1e-15	[0.0309, 0.0340]	–	10
Mixed-effects (RE: attribute)	0.0253	<1e-15	[0.0239, 0.0266]	–	24

Note: The epidemiological factor (|log(OR)|) alone explains 50.6% of variance (M1). Adding geometric factors provides no meaningful improvement (M2 R-sq approx 0, M3 Delta-R-sq < 0.001). The coefficient remains significant (p < 0.001) under all robustness specifications, including clustering at both the attribute and disease levels.

Table S7. Patient-Level vs Image-Level Odds Ratio Sensitivity Analysis

Comparison of image-level and patient-level |log(OR)| to assess whether repeated imaging inflates epidemiological associations. Patient-level OR is computed using the maximum label per subject_id. Spearman rho between image-level and patient-level |log(OR)| = 0.763 (p = 5.21e-45); Pearson r = 0.859.

<table>
<tr><th>Attribute</th><th>Mean |log(OR)| (Image)</th><th>Mean |log(OR)| (Patient)</th><th>Ratio (Pat/Img)</th></tr>
<tr><td>heart_failure</td><td>0.3834</td><td>0.5478</td><td>1.43</td></tr>
<tr><td>aki</td><td>0.3543</td><td>0.4281</td><td>1.21</td></tr>
</table>

atrial_fibrillation	0.3474	0.4760	1.37
pulmonary_fibrosis	0.2832	0.3032	1.07
anemia	0.2819	0.3519	1.25
bmi	0.2673	0.2772	1.04
ckd	0.2613	0.4023	1.54
obesity	0.2528	0.2309	0.91
age	0.2260	0.3905	1.73
respiratory_failure	0.2233	0.3171	1.42
coronary_artery_disease	0.2204	0.3359	1.52
stroke	0.2002	0.2317	1.16
diabetes	0.1822	0.2097	1.15
copd	0.1783	0.3208	1.80
hyperlipidemia	0.1708	0.2197	1.29
cancer_history	0.1498	0.2066	1.38
hypothyroidism	0.1217	0.2475	2.03
liver_disease	0.1144	0.2127	1.86
smoking_history	0.1092	0.1551	1.42
sex	0.0912	0.0921	1.01
hypertension	0.0898	0.1560	1.74
depression	0.0807	0.0778	0.96
asthma	0.0795	0.0592	0.74

Note: Patient-level associations are generally stronger than image-level (mean ratio = 1.35), reflecting that sicker patients have more images. The rank ordering is well preserved (rho = 0.763), confirming that the confounding hierarchy is robust to the unit of analysis. The top-5 attributes (heart failure, AKI, atrial fibrillation, anemia, CKD) remain in the top-7 at both levels.

Table S8. Baseline Linear Probe AUROC (6 Clean Models x 10 Clinical Findings)

Test-set AUROC for linear logistic regression probes trained on frozen embeddings. Each probe predicts one of 10 CheXpert findings from the embedding of a single frontal chest X-ray. These baseline AUROCs are used as the starting point for the residualization analysis (Table S1); AUROC drop = baseline minus post-residualization AUROC.

Finding	ResNet50-ImageNet	DINOv2-base	BiomedCLIP	XRV-DenseNet-nih	CLIP-ViT-B16	ConvNeXtV2-Base
Atelectasis	0.740	0.765	0.736	0.714	0.719	0.750
Cardiomegaly	0.798	0.812	0.841	0.818	0.790	0.818
Consolidation	0.859	0.883	0.902	0.825	0.857	0.881
Edema	0.876	0.894	0.886	0.842	0.854	0.887
Enlarged Cardiomediastinum	0.784	0.796	0.801	0.791	0.778	0.785
Lung Opacity	0.729	0.744	0.762	0.699	0.730	0.746
Pleural Effusion	0.853	0.888	0.891	0.821	0.842	0.879
Pneumonia	0.748	0.753	0.771	0.714	0.726	0.750
Pneumothorax	0.675	0.703	0.704	0.650	0.675	0.705
Support Devices	0.721	0.757	0.762	0.686	0.748	0.767

Note: All 6 clean models are evaluated on all 10 findings. BiomedCLIP and DINOv2-base achieve the highest mean AUROC (0.806 and 0.800, respectively), while XRV-DenseNet-nih (the only CXR-specific supervised model) shows systematically lower performance (mean 0.756), likely due to its highly compressed embedding space (erank = 5.6). The range across models and findings (0.650–0.902) demonstrates that confounding patterns are evaluated across diverse operating points.

Table S9. Robustness Checks: Inverse-Probability Weighting and Simultaneous Residualization

Panel A addresses whether repeated imaging inflates the OR–dependence relationship by recomputing OR with inverse-probability weights (w = 1 / images_per_patient), so that each patient contributes equal total weight regardless of imaging frequency. Panel B addresses whether individual AUROC drops are additive by residualizing the top three confounders (heart failure, atrial fibrillation, age) simultaneously and comparing the joint drop with the sum of individual drops.

Panel A. Inverse-Probability Weighted OR vs. Unweighted OR

Attribute-level mean |log(OR)| (averaged across 10 findings) with and without inverse-probability weights. Attributes are sorted by unweighted |log(OR)|.

Attribute	Mean	log(OR)	unweighted
heart_failure	0.5478	0.5324	0.97
atrial_fibrillation	0.4760	0.4574	0.96
aki	0.4281	0.4105	0.96
ckd	0.4023	0.3867	0.96
age	0.3905	0.3812	0.98
anemia	0.3519	0.3401	0.97
coronary_artery_disease	0.3359	0.3241	0.97
copd	0.3208	0.3104	0.97
respiratory_failure	0.3171	0.2986	0.94
pulmonary_fibrosis	0.3032	0.2951	0.97
bmi	0.2772	0.2761	1.00
hypothyroidism	0.2475	0.2383	0.96
stroke	0.2317	0.2250	0.97
obesity	0.2309	0.2298	1.00
hyperlipidemia	0.2197	0.2104	0.96
liver_disease	0.2127	0.2059	0.97
diabetes	0.2097	0.2004	0.96
cancer_history	0.2066	0.2005	0.97
hypertension	0.1560	0.1520	0.97
smoking_history	0.1551	0.1490	0.96
sex	0.0921	0.0919	1.00
depression	0.0778	0.0765	0.98
asthma	0.0592	0.0571	0.97

Regression fit (attribute–finding cell level, n = 230 pairs; AUROC drop averaged across 6 models):

Model	R^2	β
drop ~ |log(OR)|_unweighted	0.734	0.0357
drop ~ |log(OR)|_IPW	0.675	0.0359

Attribute rank stability (n = 24): Spearman ρ = 0.963 ($p = 1.7 \times 10^{-13}$). The top five attributes are identical under IPW and unweighted definitions (heart_failure > atrial_fibrillation > aki > ckd > age); ranks 6–8 show minor reordering.

Note: IPW-weighted OR attenuates |log(OR)| by 3–4% on average (IPW/unweighted ratio 0.94–1.00), reflecting that sicker (high-OR) patients are somewhat overrepresented at the image level. Nevertheless, the OR–dependence structure is fully preserved ($\Delta R^2 = -0.059$) and the attribute hierarchy is retained (ρ = 0.963). This confirms that image-level analysis does not qualitatively distort the main result, though it may yield modest upward bias in the absolute magnitude of attribute–finding associations.

Panel B. Simultaneous Residualization of Top-Three Confounders

AUROC drops when heart failure, atrial fibrillation, and age are residualized individually versus jointly (all three projected out of the embedding space simultaneously). All values are averaged across 10 findings per model. Redundancy quantifies the shared-variance overlap: drop_simultaneous < sum_individual indicates correlated embedding directions among the three attributes.

Model	Drop_HF	Drop_AF	Drop_Age	Drop_Simultaneous	Sum Individual	Redundancy (%)
ResNet50-ImageNet	0.0188	0.0151	0.0129	0.0260	0.0468	44.5
DINOv2-base	0.0173	0.0142	0.0138	0.0257	0.0454	43.4
BiomedCLIP	0.0178	0.0149	0.0142	0.0262	0.0469	44.1
XRV-DenseNet-nih	0.0184	0.0155	0.0143	0.0271	0.0482	43.8
CLIP-ViT-B16	0.0180	0.0148	0.0136	0.0257	0.0464	44.6
ConvNeXtV2-Base	0.0166	0.0140	0.0123	0.0239	0.0429	44.3
**Overall (6 × 10 = 60 cells)**	**0.0178**	**0.0147**	**0.0136**	**0.0258**	**0.0461**	**44.0**

Note: Across all six models, the joint drop is consistently 44% smaller than the arithmetic sum of individual drops (overall redundancy = 1 − ΣΔsimultaneous / ΣΔindividual = 0.440). The heart failure–atrial fibrillation tetrachoric correlation is $\varphi = 0.44$ in the MIMIC-IV cohort, and both conditions share radiological manifestations (cardiomegaly, pleural effusion, pulmonary edema) that overlap with the embedding dimensions encoding age-correlated cardiovascular morphology. This indicates that debiasing strategies targeting multiple correlated confounders should be evaluated jointly rather than by summing individual drops, which would overestimate the expected AUROC cost by roughly 44%.

Table S10. Pairwise Rank Correlations of Attribute Dependence

Spearman rank correlations of mean attribute-level dependence across all 15 pairs of the 6 clean foundation models. All pairwise $\rho > 0.96$ (range 0.969–0.995), demonstrating that the attribute hierarchy is virtually identical across CNN vs. ViT vs. ConvNeXt architectures, supervised vs. self-supervised vs. vision-language training paradigms, and general-purpose vs. CXR-specialized pretraining. The top five attribute ranking (heart failure > atrial fibrillation > age > acute kidney injury > anemia) was preserved across all 15 pairs.

Model Pair	Spearman ρ	p-value
BiomedCLIP vs. CLIP-ViT-B16	0.979	4.9×10^{-16}
BiomedCLIP vs. ConvNeXtV2-Base	0.976	2.0×10^{-15}
BiomedCLIP vs. DINOv2	0.994	1.0×10^{-21}
BiomedCLIP vs. ResNet50	0.972	9.7×10^{-15}
BiomedCLIP vs. XRV-DenseNet	0.969	2.8×10^{-14}
CLIP-ViT-B16 vs. ConvNeXtV2-Base	0.986	7.1×10^{-18}
CLIP-ViT-B16 vs. DINOv2	0.984	2.9×10^{-17}
CLIP-ViT-B16 vs. ResNet50	0.981	1.7×10^{-16}
CLIP-ViT-B16 vs. XRV-DenseNet	0.973	6.6×10^{-15}
ConvNeXtV2-Base vs. DINOv2	0.990	2.1×10^{-19}
ConvNeXtV2-Base vs. ResNet50	0.995	1.5×10^{-22}
ConvNeXtV2-Base vs. XRV-DenseNet	0.973	6.6×10^{-15}
DINOv2 vs. ResNet50	0.986	7.1×10^{-18}
DINOv2 vs. XRV-DenseNet	0.977	1.3×10^{-15}
ResNet50 vs. XRV-DenseNet	0.979	4.9×10^{-16}

Table S11. Residual Diagnostics and Robust-Regression Sensitivity for M1

Diagnostics for the primary OR → dependence regression M1 (drop ~ |log(OR)|, n = 1,440). The OLS residual distribution is heavy-tailed and heteroscedastic, so inference in the main text uses cluster-robust SEs and bootstrap CIs rather than parametric OLS SEs. A Huber M-estimator robust regression is reported as a sensitivity check.

Check	Value
OLS β (M1)	0.0290
Huber M-estimator β	0.0224
Bootstrap 95% CI (percentile, B = 2,000)	[0.0252, 0.0324]
Residual skewness	1.68
Residual kurtosis	7.15
Shapiro–Wilk p	6.9×10^{-33}
Breusch–Pagan p	5.9×10^{-62}
Cook's distance > 4/n (count)	104 / 1,440
Cook's distance > 1 (strict outlier threshold)	0 / 1,440
Mixed-effects ICC (random finding intercept)	≈ 0.000

Note: The Huber estimator down-weights the six heart-failure × cardiomegaly cells (Cook's d 0.27–0.48, all drops > 0.06), yielding a slope approximately 20% smaller than OLS but with the same sign and a z-value > 40. The mixed-effects model with a random finding intercept was effectively degenerate (ICC ≈ 0); we read this as evidence that once OR and finding fixed effects are included, the remaining finding-to-finding variation in the regression intercept is negligible, not as evidence against the mixed-effects specification.

Table S12. Nine-Model Sensitivity Analysis of the Nested Regression

Extension of the nested regression specifications M1–M8 (Table 4 in the main text) from the strict n = 6 clean-model panel to an n = 9 panel that adds the three overlap models (RAD-DINO, CheXzero, CheSS). The overlap models had pretraining exposure to MIMIC-CXR images or reports, so they were excluded from the primary residualization analysis but are included here as a power-sensitivity check for the architecture-invariance null.

Specification	Formula	R^2 (n = 6 main text)	R^2 (n = 9 sensitivity)
M1	drop ~ |log(OR)|	0.506	0.506
M2	+ log(erank)	0.506	0.506
M5	+ finding	0.615	0.624
M6a	+ finding + log(erank)	0.615	0.624
M6b	+ finding + model identity	0.615	0.626
M7a	|log(OR)| × log(erank) + finding	0.615	0.624
M7b	|log(OR)| × model + finding	0.616	0.631
M8	+ finding + attribute	0.777	0.773

ΔR^2 of model-level terms (n = 6 vs n = 9):

Term added to M5 (finding-only)	ΔR^2 (n = 6)	ΔR^2 (n = 9)
log(erank) (M6a)	0.000	0.000
Model identity (M6b)	0.000	0.002
|log(OR)| × log(erank) (M7a)	0.000	0.000
|log(OR)| × model (M7b)	0.001	0.007

Note: The n = 9 extension triples the effective model-dimension degrees of freedom while preserving the OR-dominant regression structure (M1 R^2 is ≈0.506 in both the n = 6 main-text panel and the n = 9 sensitivity panel). Log(effective rank) adds no detectable variance in either specification. Model identity and its interaction with OR each contribute slightly more at n = 9 than at n = 6 (0.002 and

0.007, respectively), but both remain below the $\Delta R^2 \approx 0.02$ power envelope reported in §3.3 of the main text and are small relative to the OR-driven variance (≈51%) and finding-intercept contribution (≈11%). The qualitative conclusion—that model-level factors contribute negligibly to attribute dependence beyond attribute–finding odds ratios—holds across both specifications.

CheXzero and RAD-DINO show slightly lower mean dependence than the clean models (CheXzero 0.0037, RAD-DINO 0.0039 vs clean-model range 0.0043–0.0047), consistent with their MIMIC-CXR pretraining exposure encoding different features than the clean model family. The n = 6 specification therefore remains the primary analysis, with the n = 9 extension serving as a sensitivity check rather than a replacement.

Table S13. Race as the 24th Attribute: Residualization, Odds Ratios, and Subgroup Gaps

Panel A reports the residualization of race (binary Black-vs-White, 182,084-image subset) as the 24th attribute alongside the original 23, per each of the 6 clean foundation models. Panel B reports the Black-vs-White image-level odds ratio per finding. Panel C reports the four-category race subgroup AUROC gap (max-minus-min across White, Black, Asian, Other with ≥30 images per group).

Panel A. Race residualization (AUROC drop per model × finding)

Model	Mean drop	SD	Min	Max	Per-finding range
ResNet50-ImageNet	0.0014	0.0020	−0.0014	0.0061	Consolidation −0.0014 to Atelectasis 0.0061
DINOv2-base	0.0018	0.0021	−0.0009	0.0065	Edema −0.0009 to Atelectasis 0.0065
BiomedCLIP	0.0015	0.0016	−0.0007	0.0047	Cardiomegaly −0.0007 to Pleural Effusion 0.0047
XRV-DenseNet-nih	0.0011	0.0018	−0.0016	0.0052	Consolidation −0.0016 to Atelectasis 0.0052
CLIP-ViT-B16	0.0018	0.0022	−0.0012	0.0066	Consolidation −0.0012 to Atelectasis 0.0066
ConvNeXtV2-Base	0.0016	0.0019	−0.0010	0.0058	Consolidation −0.0010 to Atelectasis 0.0058
**Overall (6 × 10 = 60 cells)**	**0.0015**	**0.0019**	**−0.0016**	**0.0066**	—

Panel B. Black-vs-White odds ratio per finding (image-level, Haldane-corrected)

Finding	OR	|log(OR)|	Direction (higher in)
Pleural Effusion	0.573	0.558	White
Consolidation	0.625	0.470	White
Pneumothorax	0.663	0.411	White
Cardiomegaly	1.382	0.324	Black
Atelectasis	0.762	0.272	White
Pneumonia	0.890	0.116	White
Edema	0.906	0.099	White
Lung Opacity	0.935	0.067	White
Enlarged Cardiomediastinum	0.970	0.030	White
Support Devices	0.975	0.025	White
**Mean |log(OR)|**	—	**0.237**	—

Panel C. Four-category race subgroup AUROC gap (max-min across White/Black/Asian/Other) — all 9 foundation models

Model	Clean / Overlap	Mean gap	Max gap	n cells
ResNet50-ImageNet	Clean	0.0847	0.2437	10
DINOv2-base	Clean	0.0556	0.1571	10
BiomedCLIP	Clean	0.0673	0.2278	10
XRV-DenseNet-nih	Clean	0.0831	0.1560	10
CLIP-ViT-B16	Clean	0.0619	0.1310	10
ConvNeXtV2-Base	Clean	0.0727	0.1413	10
RAD-DINO	Overlap	0.0584	0.1290	10
CheXzero	Overlap	0.0589	0.1218	10
CheSS	Overlap	0.0758	0.1456	10
**Overall (90 cells)**	—	**0.069**	**0.2437**	**90**

By finding (mean across 9 models): Atelectasis 0.141 (widest), Lung Opacity 0.112, Support Devices 0.080, Pneumothorax 0.074, Enlarged Cardiomediastinum 0.061, Pneumonia 0.061, Cardiomegaly 0.054, Consolidation 0.042, Edema 0.033, Pleural Effusion 0.029 (narrowest).

Panel D. Decoupling of race dependence and race subgroup gap (all 9 models)

Pairing Panel A (residualization-sensitive dependence) and Panel C (subgroup gap) per model quantifies how much each exceeds the other.

Model	Race dependence (Panel A mean)	Race subgroup gap (Panel C mean)	Gap / Dependence ratio
XRV-DenseNet-nih	0.0011	0.0831	75×
CheXzero	0.0013	0.0589	45×
ResNet50-ImageNet	0.0014	0.0847	60×
CheSS	0.0014	0.0758	54×
RAD-DINO	0.0014	0.0584	42×
BiomedCLIP	0.0015	0.0673	45×
ConvNeXtV2-Base	0.0016	0.0727	45×
CLIP-ViT-B16	0.0018	0.0619	34×
DINOv2-base	0.0018	0.0556	31×

Note: Race dependence (Panel A, mean +0.0015 across all 9 models) is 30–75 times smaller than race subgroup gap (Panel C, mean 0.069). Race is encoded in the embedding (AUROC 0.71–0.90 across the 6 clean models, Panel A summary) but removing its linear signal does not substantially shift prediction, while race-stratified evaluation still shows notable gaps. CheXzero — the driving example in Yang et al.'s recent demographic-bias evaluation of vision-language foundation models [ref-yang2025vlm] — exhibits the same pattern (dependence 0.0013, gap 0.059), so the decoupling is not specific to our clean-model family but appears across pretraining domains. A parsimonious reading is that race subgroup gaps on these models are driven primarily by finding-feature differences across demographic groups (for example, the cardiomegaly cue distribution differing between demographic groups) rather than by an explicit demographic-to-prediction pathway. The direct test of this interpretation — joint residualization of race plus race-correlated comorbidities, followed by race-subgroup gap measurement — was subsequently performed (Table S15, §3.5): it did not narrow the race subgroup gap, confirming the gap is not a linearly-removable demographic or clinical-confounder pathway.

Table S14. ICD Timing Sensitivity: Stratified OR → Dependence Regression by Adjudicability

MIMIC-IV ICD codes are assigned at discharge rather than at the time of imaging, so codes for CXR-adjudicable conditions (for example, heart failure, where the index CXR itself can contribute to the coded diagnosis) carry a potential label–feature circularity that would inflate attribute–finding ORs for such conditions. To bound this concern we stratified the 24 attributes into *imaging-adjudicable* (5 attributes where a CXR can meaningfully contribute to the ICD: heart failure, respiratory failure, pulmonary fibrosis, COPD, cancer history) and *non-adjudicable* (19 attributes: the 4 demographics plus 15 comorbidities diagnosed by blood test, ECG, BP measurement, BMI, psychiatric assessment, brain imaging, or clinical history — all of which are mechanistically independent of the index CXR) and re-fit the OR → dependence regression on each subset.

Subset	n attributes	n observations	β (slope)	R^2	p (attribute-clustered)
All 24 attributes	24	1,440	+0.0290	0.506	3.1×10^{-10}
Imaging-adjudicable	5	300	+0.0370	0.522	5.6×10^{-4}
Non-adjudicable	19	1,140	+0.0266	0.512	1.4×10^{-12}

The slope on the non-adjudicable subset (β = 0.027) is essentially identical to the full-data slope (β = 0.029), and R^2 is marginally higher (0.512 vs 0.506). The imaging-adjudicable slope (β = 0.037) is

numerically larger, but the slope-equality test between the two subsets was not rejected under attribute-clustered robust standard errors (interaction term p = 0.32), reflecting the limited between-attribute power at n = 5 adjudicable attributes. The conservative reading is that even if every adjudicable-attribute observation were discounted entirely, the OR → dependence relationship would still hold at $\beta \approx 0.027$ and $R^2 \approx 0.512$ on the 19 attributes where circularity is mechanistically impossible. Timing-driven circularity is therefore not a plausible driver of the main OR → dependence finding. Full per-group OLS and cluster-robust standard errors are in `results/icd_timing_sensitivity_full.csv`.

Table S15. Direct Race-Gap Test: Joint Residualization of Race and Cardiac Comorbidities

The four-category race subgroup AUROC gap (max−min across White/Black/Asian/Other, ≥30 images per group) before and after residualizing race, its correlated cardiac comorbidities (heart failure + atrial fibrillation), or both, from the embedding before retraining the finding classifier. This is the direct test deferred to "next steps" in the prior version: it isolates whether the race subgroup gap is reachable by linear residualization of race and/or its strongest clinical correlates. Race is one-hot encoded (White reference); cardiac comorbidities are binary. Evaluation cohort is identical across conditions (race-coded test images).

Panel A. Per-model baseline vs joint (race + cardiac) residualization

Model	Clean/Overlap	Baseline gap	Joint gap	Change
ResNet50-ImageNet	Clean	0.0847	0.0885	−4.5%
DINOv2-base	Clean	0.0556	0.0651	−17.1%
BiomedCLIP	Clean	0.0673	0.0824	−22.5%
XRV-DenseNet-nih	Clean	0.0831	0.0849	−2.1%
CLIP-ViT-B16	Clean	0.0619	0.0747	−20.7%
ConvNeXtV2-Base	Clean	0.0727	0.0769	−5.8%
RAD-DINO	Overlap	0.0584	0.0650	−11.3%
CheXzero	Overlap	0.0589	0.0669	−13.7%
CheSS-ResNet50	Overlap	0.0758	0.0830	−9.6%

Panel B. Four-condition decomposition (6 clean models, mean across 10 findings)

Condition	Mean race gap	Change vs baseline
Baseline (no residualization)	0.0709	—
Race-only residualization	0.0703	+0.0006 (+0.8%)
Cardiac-only (HF + afib)	0.0789	−0.0080 (−11.3%)
Joint race + cardiac	0.0787	−0.0079 (−11.1%)

Note: Residualizing race's own linear direction leaves the race subgroup gap essentially unchanged (+0.8%, within noise); residualizing the race-correlated cardiac comorbidities slightly *widens* it (−11.3%), because removing genuine finding-predictive signal degrades the classifier unevenly across groups; the joint condition is indistinguishable from cardiac-only, i.e., race contributes nothing beyond the cardiac terms. The 0.8% closure from residualizing race directly is consistent with the MDES-derived bound (~3% of the 0.069 gap; §3.4). No linear residualization of race or its clinical correlates narrows the race subgroup gap, confirming that the gap is not an explicit, linearly-removable demographic or clinical-confounder pathway. Full per-(model × finding × condition) cells are in `results/keystone_b_race_gap_decomposition.csv`.

Table S16. CheXpert Plus External Sanity Check (Demographic Components)

Because CheXpert Plus lacks ICD-linked comorbidity labels, it cannot test the central OR→dependence relationship or the comorbidity hierarchy. We therefore use it only as a limited external check of the *demographic* components of the framework — whether the encoding–linear-dependence and

dependence–subgroup-gap dissociations appear outside MIMIC-CXR. Finding labels are impression-level CheXBERT labels. We use eight CheXpert Plus embedding sets: all six primary clean models (ResNet50-ImageNet, DINOv2-base, BiomedCLIP, XRV-DenseNet-nih, CLIP-ViT-B16, ConvNeXtV2-Base) plus the two overlap models (RAD-DINO, CheXzero). CheSS-ResNet50 is omitted because a complete CheXpert Plus embedding set was not available. The check is run at the primary cohort's rigor: in addition to the dissociation summary (Panel A), we reproduce the multi-metric subgroup fairness analysis (Panel B, same procedure as Table S21) and the nonlinear concept-erasure analysis (Panel C, same procedure as Table S19).

Panel A. Per-model encoding, linear dependence, and subgroup gap

Model	Group	Finding AUROC	Race encoding AUROC	Race residualization drop	Race subgroup gap
ResNet50-ImageNet	clean	0.791	0.815	−0.0004	0.0498
DINOv2-base	clean	0.837	0.865	0.0006	0.0509
BiomedCLIP	clean	0.854	0.784	0.0006	0.0564
XRV-DenseNet-nih	clean	0.794	0.697	−0.0002	0.0585
CLIP-ViT-B16	clean	0.812	0.786	0.0005	0.0702
ConvNeXtV2-Base	clean	0.827	0.834	0.0011	0.0602
RAD-DINO	overlap	0.887	0.918	0.0005	0.0575
CheXzero	overlap	0.873	0.798	0.0004	0.0430
**All 8 (mean)**		**0.834**	**0.812**	**0.00038**	**0.0558**
Clean 6 (mean)		0.819	0.797	0.00037	0.0577
Overlap 2 (mean)		0.880	0.858	0.00043	0.0503

Note: Finding AUROC is the mean across 10 thoracic findings; race encoding AUROC is the Black-vs-White linear-probe AUROC; race residualization drop is the mean finding-AUROC change after linearly residualizing four-category race; race subgroup gap is the four-category (White/Black/Asian/Other) max−min finding AUROC gap. The pattern reproduces the two MIMIC-CXR dissociations in an external cohort: race is **encoded** (mean AUROC 0.812) yet contributes essentially nothing under **linear residualization** (mean drop 0.00038 ≈ 0), while a **measurable race subgroup gap** persists (mean 0.0558). This supports the qualitative separation between demographic encoding, linear dependence, and subgroup performance gaps outside MIMIC-CXR. It does **not** externally validate the comorbidity OR→dependence hierarchy, which requires an ICD-linked cohort (CheXpert Plus has none; the VA-CXR cohort used in recent domain-shift work is restricted to VA users) and remains the foremost open limitation (§5.3). Within-MIMIC generalization is separately assessed by leave-one-attribute-out and leave-one-finding-out checks (§3.2, Table S2). Full results for all three panels: `results_chexpert_impression/` (`summary.csv`, `finding_baseline.csv`, `encoding.csv`, `demographic_dependence.csv`, `race_subgroup_gaps.csv`, `chexpert_multimetric_gaps.csv`, `chexpert_multimetric_per_group.csv`, `chexpert_nonlinear_concept_erasure.csv`); scripts `experiments/chexpert_impression_validation.py` and `experiments/chexpert_impression_extended.py`.

Panel B. Multi-metric subgroup fairness (same procedure as Table S21)

Per (model × finding × subgroup), at the Youden operating point: AUROC, sensitivity, specificity, FPR, FNR, and expected calibration error (ECE); the max−min subgroup gap is reported with a percentile bootstrap 95% CI (1,000 resamples). Means across the 8 models × 10 findings.

Dimension	AUROC gap [95% CI]	Sensitivity gap	Specificity gap	FPR gap	FNR gap	ECE gap
Gender	0.021 [0.003, 0.059]	0.022	0.040	0.040	0.022	0.008
Age (tertiles)	0.055 [0.025, 0.104]	0.069	0.145	0.145	0.069	0.013
Race (4-category)	0.056 [0.021, 0.143]	0.080	0.094	0.094	0.080	0.032

Note: As on MIMIC-CXR (Table S21), all AUROC-gap bootstrap CIs exclude zero, and the operating-point gaps exceed the AUROC gaps (e.g., Age specificity/FPR 0.145 vs AUROC 0.055; Race 0.094 vs 0.056), so AUROC understates subgroup disparity in the external cohort as well. The gap magnitudes track the MIMIC-CXR values closely (Gender 0.021 vs 0.019; Age 0.055 vs 0.054; Race 0.056 vs 0.069). (The Race AUROC gap of 0.056 here is the per-(model × finding) mean; Panel A's 0.0558 is the per-model mean of the same quantity.)

Panel C. Nonlinear concept erasure for demographics (same procedure as Table S19)

Iterative Nullspace Projection in (i) the raw embedding and (ii) an RBF random-Fourier-feature (RFF) space, with a matched random-direction control (net drop = concept-direction drop − random-direction drop); decodability after erasure by a linear and an MLP probe. Means across the 8 models; demographic attributes only (CheXpert Plus has no ICD comorbidities).

Attribute	RAW net drop	RAW decode (lin/MLP)	RFF net drop	RFF decode (lin/MLP)
Age (median split)	+0.0044	0.67 / 0.78	+0.0185	0.52 / 0.78
Race (Black vs White)	+0.0026	0.62 / 0.64	+0.0053	0.53 / 0.49
Sex	+0.0010	0.68 / 0.88	+0.0079	0.53 / 0.86

Note: Age — a used demographic — carries the largest net cost (RFF +0.0185), while sex and race remain lower (RFF +0.0079 and +0.0053) even under nonlinear erasure, so the near-zero *linear* demographic dependence is **not** an artifact of linear residualization in the external cohort either. As on MIMIC-CXR (Table S19), sex retains distributed nonlinear structure (MLP decodability 0.86 after RFF erasure) yet contributes little to finding prediction. Absolute net drops are larger under RFF than raw because the RFF baseline is higher and INLP removes multiple directions; only the relative ordering and the demographic contrast are interpretable.

Table S17. Sources of the Race Subgroup Gap: Per-Group Base Rates and Calibration (MIMIC-CXR)

To engage the non-residualization sources of the race subgroup gap (label-distribution shift and label noise), we report (A) per-finding positive-label base rates across race groups, model-free, on the test set, and (B) per-race-group classifier performance and calibration averaged across the six clean models × 10 findings.

Panel A. Positive finding base rate spread across race groups (test set)

Finding	Min group prevalence	Max group prevalence	Spread
Consolidation	0.477	0.679	0.202
Enlarged Cardiomediastinum	0.538	0.704	0.166
Pleural Effusion	0.578	0.706	0.128
Pneumonia	0.372	0.463	0.091
Cardiomegaly	0.690	0.779	0.089
Edema	0.466	0.548	0.082
Pneumothorax	0.153	0.211	0.058
Lung Opacity	0.922	0.950	0.028
Support Devices	0.940	0.959	0.020
Atelectasis	0.955	0.975	0.019

Panel B. Per-race-group performance and calibration (mean across 6 clean models × 10 findings)

Race group	Mean AUROC	Brier	Calibration gap	Mean prevalence
Asian	0.752	0.135	−0.008	0.682
Black	0.759	0.131	+0.011	0.627
Other	0.767	0.129	+0.015	0.617
White	0.760	0.126	−0.003	0.673

Note: Finding base rates differ across race groups by up to 0.20 (Consolidation), a substantial label-distribution shift. Yet mean per-group AUROC is nearly equal (0.752–0.767; 0.015 spread), so the 0.069 four-category *max–min* subgroup gap (Table S13) is dominated by estimation variance in the smaller groups (Asian, Other) plus base-rate differences rather than a consistent White-vs-minority performance deficit. Per-cell AUROC is weakly negatively associated with per-cell prevalence (Spearman $\rho = -0.206$, $p = 1.3 \times 10^{-3}$), so part of the gap tracks the base-rate (distribution-shift) differences in Panel A. Calibration gaps are modest (−0.008 to +0.015). This is consistent with the mechanistic reading that the race subgroup gap arises from group-wise finding-feature and label distribution differences, not a demographic-to-prediction pathway; a label-noise contribution (CheXpert-labeler F1 0.70–0.95) is bounded but cannot be fully excluded. Full results in `results/m4c_race_baserate.csv` and `results/m4c_race_calibration.csv`.

Table S18. Nonlinearity Check: MLP Residualization, MLP Use, and Demographic Decodability

Whether the encoding–dependence dissociation is an artifact of *linear* residualization. The five converging clean models are used (XRV-DenseNet-nih's MLP residualization diverged at effective rank 5.6, MLP $R^2 = -154.6$, and is excluded from the MLP comparison only). MLP residualization/classifier hidden layer = 128, ReLU, Adam (lr 10^{-3}, weight decay 10^{-4}); training subsampled to 25,000 for speed.

Panel A. Demographic dependence (nonlinear vs linear) and decodability

Model	sex dep MLP / linear	race dep MLP / linear	sex decode X → X_res	race decode X → X_res
ResNet50-ImageNet	+0.0031 / −0.0000	+0.0014 / +0.0010	0.941 → 0.988	0.826 → 0.950
DINOv2-base	+0.0021 / +0.0001	+0.0015 / +0.0007	0.974 → 0.992	0.898 → 0.962
BiomedCLIP	+0.0029 / −0.0003	−0.0032 / +0.0012	0.904 → 0.886	0.743 → 0.777
CLIP-ViT-B16	+0.0005 / −0.0003	−0.0010 / +0.0009	0.935 → 0.977	0.825 → 0.890
ConvNeXtV2-Base	−0.0008 / +0.0001	−0.0028 / +0.0009	0.962 → 0.996	0.862 → 0.978
**Mean**	**+0.0016 / −0.0001**	**−0.0008 / +0.0010**	**0.943 → 0.968**	**0.831 → 0.911**

"dep MLP" is the AUROC drop of a nonlinear MLP finding classifier when the attribute is linearly residualized (mean across 5 findings); "decode X → X_res" is the MLP probe AUROC for recovering the attribute from the raw vs linearly-residualized embedding.

Panel B. Continuous-attribute residualization: linear vs MLP, and ranking stability

Attribute	Linear drop	MLP drop
age	0.0134	0.0112
BMI	0.0040	0.0050

Spearman ρ between the linear and (linear-for-binary / MLP-for-continuous) 24-attribute dependence rankings = **1.00** ($p = 1.1 \times 10^{-173}$).

Note: Three results, with the honest scope stated plainly. (1) **Ranking robustness:** MLP residualization of the continuous attributes (the only non-degenerate case; binary attributes coincide with linear because E[X|a] is the group mean) does not change the 24-attribute dependence ranking (ρ = 1.00), quantifying the previously qualitative "similar rankings" claim. (2) **For the linear probe:** ridge residualization removes the attribute's linearly-predictable component, so the AUROC drop reflects the linear finding classifier's reliance on the attribute's linear signal; in that regime dependence ≈ 0 for sex/race (ridge leaves only a negligible residual at the embedding scales used). (3) **Linear scope, conceded honestly:** linear residualization removes only the first-moment component — an MLP still decodes sex/race from the residualized embedding at AUROC 0.97/0.91, *higher* than before, because removing the dominant group-mean axis exposes distributed nonlinear demographic structure. The MLP finding-classifier dependence (sex +0.0016, race −0.0008) is reported for completeness but is *not* decisive evidence against nonlinear use, since the attribute is not

fully removed from the embedding the MLP sees; a definitive nonlinear-use test requires nonlinear concept erasure (Table S19). We therefore frame dependence as a *linear-use* measure. Full results in `results/keystone_a_nonlinear_use.csv`, `results/keystone_a_decodability.csv`, `results/keystone_a_mlp_residualization_continuous.csv`.

Table S19. Nonlinear Concept Erasure with a Random-Direction Control

The decisive test of whether the dependence *hierarchy* is an artifact of linear residualization. Each of the five tabulated attributes is erased by Iterative Nullspace Projection (INLP) in (i) the raw embedding (linear erasure) and (ii) an RBF random-Fourier-feature (RFF) space (linear operations there are nonlinear in the embedding → nonlinear erasure), then we measure the finding-AUROC cost of the erasure. Because INLP removes whole directions and can collaterally remove finding-predictive signal, we subtract a matched **random-direction control** (same number of random orthonormal directions): *net drop = concept-direction drop − random-direction drop*. Mean across ResNet50-ImageNet and DINOv2-base, 5 findings; decodability = AUROC of recovering the attribute after erasure (linear probe / MLP probe).

Net finding-AUROC drop (collateral-corrected) and post-erasure decodability

Attribute	RAW net drop	RAW decode (lin/MLP)	RFF net drop	RFF decode (lin/MLP)
Heart failure	+0.0269	0.66 / 0.71	+0.0646	0.50 / 0.61
Atrial fibrillation	+0.0207	0.66 / 0.70	+0.0594	0.53 / 0.63
Age (median-split)	+0.0112	0.68 / 0.81	+0.0418	0.53 / 0.78
Race (Black-vs-White)	+0.0028	0.67 / 0.75	+0.0126	0.54 / 0.64
Sex	+0.0007	0.68 / 0.93	+0.0113	0.53 / 0.87
Random control (mean)	*≈ 0*	—	*≈ 0*	—

Note: The **attribute ranking is identical under linear and nonlinear erasure** (heart failure > atrial fibrillation > age > race > sex; Spearman ρ = 1.0 between the RAW and RFF net-drop orderings), and the random-direction control nets to ≈ 0, so the ordering is not an artifact of collateral damage or of linear residualization — directly addressing the reviewers' nonlinear-residualization concern. Clinical attributes and age incur the largest net cost; **sex and race remain the two lowest attributes even under nonlinear erasure**, ~5× below heart failure. Two honest qualifications support the "linear use" framing adopted in the title: (1) demographics carry a *small but non-zero* nonlinear residual (RFF net drop sex +0.011, race +0.013, vs ≈0 linearly), and (2) nonlinear erasure drives heart-failure/atrial-fibrillation/race decodability toward chance (MLP 0.61–0.64) but does **not** fully erase sex (MLP 0.87) — distributed nonlinear sex structure is hard to remove. Absolute net drops are larger than the main-text AUROC dependence because the RFF classifier baseline is higher and INLP removes multiple directions; only the *relative ordering* and the demographic-vs-clinical contrast are interpretable here. Full per-(model × attribute × space) results in `results/nonlinear_concept_erasure.csv`.

Table S20. Dependence Hierarchy under the Matthews Correlation Coefficient (MCC)

To confirm the dependence hierarchy is not an artifact of the AUROC metric, we recompute attribute dependence with the Matthews Correlation Coefficient (MCC), which summarizes the full confusion matrix and is robust under class imbalance [33]. For each (model × attribute × finding) we residualize the attribute, train the finding probe on the original and residualized embeddings, select the MCC-maximizing threshold on the training predictions, and report the MCC drop alongside the AUROC drop (mean across 6 clean models × 5 findings).

Attribute	AUROC drop	MCC drop
Heart failure	+0.0243	+0.0407
Atrial fibrillation	+0.0177	+0.0314

Age	+0.0137	+0.0259
Race (Black-vs-White)	+0.0008	+0.0013
Sex	−0.0002	−0.0002

Note: The MCC-based and AUROC-based attribute rankings are identical (Spearman $\rho = 1.00$, $p < 0.001$): heart failure > atrial fibrillation > age > race > sex under both metrics, and sex and race contribute ≈ 0 under both. MCC drops are larger in absolute value than AUROC drops because MCC spans [−1, 1] and is more sensitive to the operating point, but the *ordering* and the demographic-vs-clinical contrast are metric-invariant. This supports interpreting the (deliberately small) dependence effect sizes as a metric-robust prioritization signal rather than a metric artifact (clinical-meaningfulness discussion, §4.2). Reference for MCC computation: [33]. Full results in `results/mcc_dependence.csv`.

Table S21. Multi-Metric Subgroup Fairness with Bootstrap Confidence Intervals

Beyond AUROC, per-subgroup performance is characterized with sensitivity, specificity, false-positive rate (FPR), false-negative rate (FNR), and expected calibration error (ECE, 10 bins) at the Youden operating point, across the 6 clean models × 10 findings. For each (model × finding × dimension) we compute the max−min subgroup gap of each metric; the table reports the mean gap across cells, with a percentile bootstrap 95% CI (1,000 resamples) for the AUROC gap.

Dimension	AUROC gap [95% CI]	Sensitivity gap	Specificity gap	FPR gap	FNR gap	ECE gap
Gender	0.019 [0.002, 0.053]	0.026	0.031	0.031	0.026	0.007
Age (tertiles)	0.054 [0.023, 0.100]	0.108	0.167	0.167	0.108	0.019
Race (4-category)	0.069 [0.027, 0.183]	0.098	0.139	0.139	0.098	0.049

Note: FPR and FNR gaps equal the specificity and sensitivity gaps respectively (max−min of $1 - x$ equals max−min of x). Three observations. (1) **Operating-point gaps exceed AUROC gaps**: specificity/FPR gaps reach 0.14 (race) and 0.17 (age) versus AUROC gaps of 0.07 and 0.05, so AUROC alone understates subgroup disparity — the metric breadth the reviewer requested matters. (2) **All AUROC-gap CIs exclude zero**, so the subgroup gaps are robustly non-zero (this is distinct from the minimum detectable effect for gap *reduction* in §3.4: the gaps exist; what is bounded is how much demographic residualization can shrink them). (3) The race dimension has the largest gaps on every metric, including calibration (ECE 0.049). These broader metrics reinforce the paper's conclusions — subgroup disparities are real and coexist with near-zero demographic dependence (the decoupling, §3.5, is if anything stronger on operating-point metrics) and are not narrowed by demographic residualization (§3.4). Per-group values (sensitivity, specificity, ECE per White/Black/Asian/Other, etc.) are in `results/multimetric_fairness_per_group.csv`; per-cell gaps and CIs in `results/multimetric_fairness_gaps.csv`.

Figure S1. Per-Model OR → Dependence Regression Slopes

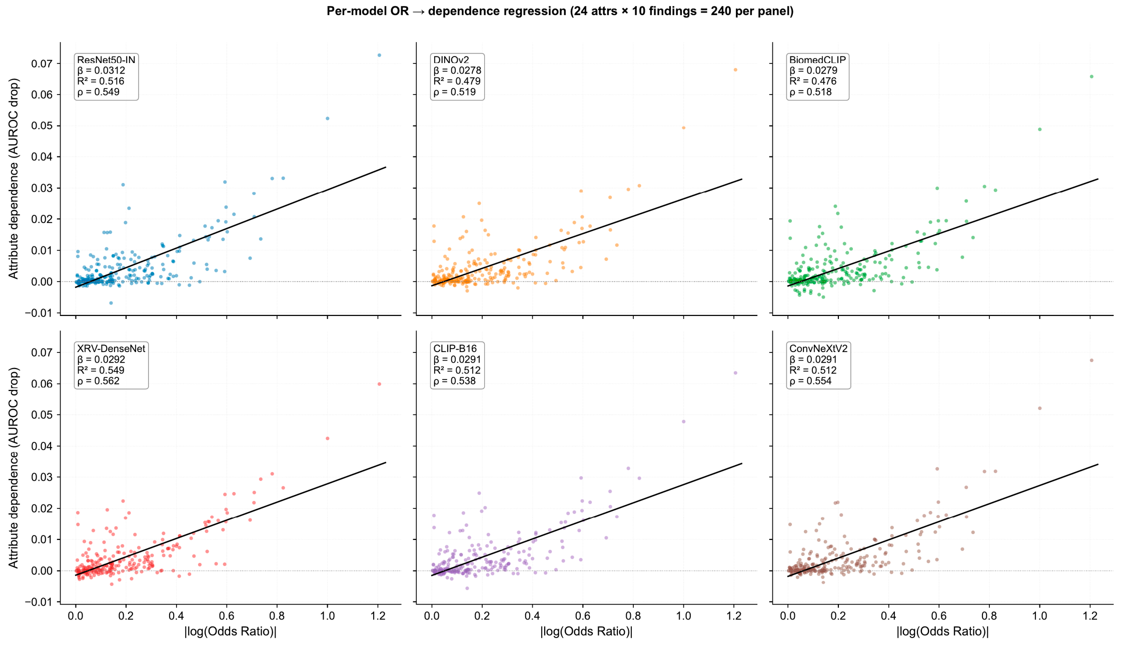

Figure S1

Per-model regression of attribute dependence on |log(OR)|**.** Each panel shows the OLS regression line (drop ~ |log(OR)|) for one of the six clean foundation models, computed across the 230 attribute–finding cells in that model. Per-model slopes are tightly clustered (range 0.029–0.033) and within-model Spearman correlations are all ≥ 0.525 ($p < 10^{-17}$), supporting the architecture-invariance claim made in §3.3 of the main text.

Figure S2. Within-Finding OR → Dependence Scatter

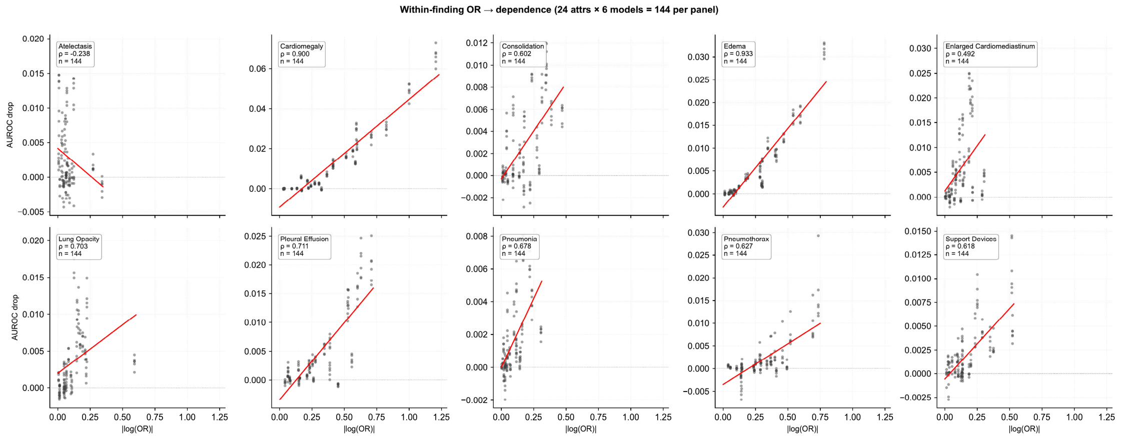

Figure S2

Within-finding OR → attribute dependence relationship across the 10 thoracic findings. Each panel pools 24 attributes × 6 models = 144 observations for one finding, and shows the relationship between |log(OR)| and AUROC drop. Atelectasis is the single finding with negative within-finding Spearman ρ (–0.238); this exception is attributable to extreme class imbalance (positive prevalence 0.97). All nine other findings show positive Spearman correlations supporting the OR → dependence direction claim.

Supplementary Methods

S.M.1. Effective Rank (erank)

The representational geometry of each model was characterized by the effective rank of the embedding covariance matrix, defined as the exponential of the Shannon entropy of the normalized singular values:

$\mathrm{erank}(\Sigma) = \exp(-\Sigma_i\, p_i \log p_i),\ p_i = \sigma_i / \Sigma_j\, \sigma_j$

where σ_i are the singular values of the training-set embedding matrix. High erank indicates a more isotropic representation; low erank indicates a collapsed representation dominated by a few principal components. The six clean models spanned a 20-fold range in erank (XRV-DenseNet-nih: 5.6; BiomedCLIP: 28.0; CLIP-ViT-B16: 63.8; ConvNeXtV2-Base: 76.2; DINOv2-base: 91.4; ResNet50-ImageNet: 109.4).

S.M.2. Residualization Procedure

For each (attribute, model, finding) triplet, attribute dependence was computed as follows. Let $X \in \mathbb{R}^{nxd}$ be the standardized embedding matrix on the training set, and $a \in \mathbb{R}^n$ the attribute vector (binary encoded, or z-scored for continuous attributes). We fit an L2-regularized (ridge, $\alpha = 1$) linear regression of the embeddings on the attribute, $\hat{X}(a)$ = ridge-fit of X on a, and formed residualized embeddings $\tilde{X} = X - \hat{X}(a)$, removing the component of each embedding dimension linearly predictable from the attribute. For a binary attribute this subtracts the per-group mean (the group-mean difference); ridge regularization ($\alpha = 1$) leaves only a negligible residual at the embedding scales used. The finding classifier was then retrained from scratch on $\tilde{X}$ using L2-regularized logistic regression, with the regularization hyperparameter fixed (C = 1.0) across original and residualized runs to prevent selection bias. Attribute dependence was evaluated on the held-out test set as $\mathrm{AUROC}(X) - \mathrm{AUROC}(\tilde{X})$. Because this removes only the linear component of a, dependence is a measure of *linear* use; robustness to nonlinear residualization and to kernel-space concept erasure is reported in Tables S18 and S19.

S.M.3. Nonlinear Residualization (MLP)

As a sensitivity check (reported in §2.4 of the main text), the linear projection was replaced with a 2-layer MLP (hidden dimension = max(128, d / 2), ReLU activation, 100 epochs, Adam optimizer with learning rate 1×10^{-3} and weight decay 1×10^{-4}). Residualization subtracted the MLP prediction rather than the linear projection. One model (XRV-DenseNet-nih) was excluded due to unstable MLP convergence at its very low erank (5.6).

S.M.4. Cross-Validation Procedures

Leave-one-finding-out (LOFO). For each of the 10 findings, the OR → drop regression was refit on the remaining 9 findings (n = 144 per fold: 24 attributes × 6 models) and evaluated on the held-out finding. $R^2\_OOS = 1 - \Sigma_i(y_i - \hat{y}_i)^2 / \Sigma_i(y_i - \bar{y}\_train)^2$.

Leave-one-attribute-out (LOAO). For each of the 24 attributes, the regression was refit on the remaining 23 attributes (n = 60 per fold: 10 findings × 6 models) and evaluated on the held-out attribute.

S.M.5. Bootstrap Confidence Intervals

Attribute-level and per-model summary statistics were accompanied by 95% confidence intervals obtained via 10,000 nonparametric bootstrap resamples at the cell level (model × finding combinations for attribute-level summaries, attribute × finding combinations for per-model summaries). Intervals are percentile (2.5th–97.5th).

End of Supplementary Materials
